# Does Reorganization of Clinicopathological Information Improve Prognostic Stratification and Prediction of Chemoradiosensitivity in Sinonasal Carcinomas? A Retrospective Study on 145 Patients

**DOI:** 10.3389/fonc.2022.799680

**Published:** 2022-06-03

**Authors:** Marco Ferrari, Davide Mattavelli, Alberto Schreiber, Tommaso Gualtieri, Vittorio Rampinelli, Michele Tomasoni, Stefano Taboni, Laura Ardighieri, Simonetta Battocchio, Anna Bozzola, Marco Ravanelli, Roberto Maroldi, Cesare Piazza, Paolo Bossi, Alberto Deganello, Piero Nicolai

**Affiliations:** ^1^ Section of Otorhinolaryngology—Head and Neck Surgery, Department of Neurosciences, University of Padua—”Azienda Ospedale Università di Padova”, Padua, Italy; ^2^ Technology for Health (PhD program), Department of Information Engineering, University of Brescia, Brescia, Italy; ^3^ Guided Therapeutics Program International Scholar, University Health Network, Toronto, Canada; ^4^ Unit of Otorhinolaryngology—Head and Neck Surgery, Department of Medical and Surgical Specialties, Radiologic Sciences, and Public Health, University of Brescia—”ASST Spedali Civili di Brescia”, Brescia, Italy; ^5^ Artificial Intelligence in Medicine and Innovation in Clinical Research and Methodology (PhD program), Department of Clinical and Experimental Sciences, University of Brescia, Brescia, Italy; ^6^ Unit of Pathology, “ASST Spedali Civili di Brescia”, Brescia, Italy; ^7^ Unit of Radiology, Department of Medical and Surgical Specialties, Radiologic Sciences, and Public Health, University of Brescia—”ASST Spedali Civili di Brescia”, Brescia, Italy; ^8^ Unit of Medical Oncology, Department of Medical and Surgical Specialties, Radiologic Sciences, and Public Health, University of Brescia—”ASST Spedali Civili di Brescia”, Brescia, Italy

**Keywords:** sinonasal, carcinoma, skull base (head and neck), classification, machine learning, prognosis, chemotherapy, radiotherapy

## Abstract

**Background:**

The classification of sinonasal carcinomas (SNCs) is a conundrum. Consequently, prognosis and prediction of response to non-surgical treatment are often unreliable. The availability of prognostic and predictive measures is an unmet need, and the first logical source of information to be investigated is represented by the clinicopathological features of the disease. The hypothesis of the study was that clinicopathological information on SNC could be exploited to better predict prognosis and chemoradiosensitivity.

**Methods:**

All patients affected by SNC who received curative treatment, including surgery, at the Unit of Otorhinolaryngology—Head and Neck Surgery of the University of Brescia between October 1998 and February 2019 were included in the analysis. The institutional series was reviewed and a survival analysis was performed. Machine learning and multivariable statistical methods were employed to develop, analyze, and test 3 experimental classifications (classification #1, based on cytomorphological, histomorphological, and differentiation information; classification #2, based on differentiation information; and classification #3, based on locoregional extension) of SNC, based on the inherent clinicopathological information. The association of experimental classifications with prognosis and chemoradiosensitivity was tested.

**Results:**

The study included 145 patients. From a prognostic standpoint, the machine learning-generated classification of SNC provided better prediction than the current World Health Organization classification. However, the prediction of the chemoradiosensitivity of SNC was not achievable.

**Conclusions:**

Reorganization of clinicopathological information, with special reference to those related to tumor differentiation, can improve the reliability of prognosis of SNC. Prediction of chemoradiosensitivity remains an unmet need and further research is required.

## Introduction

Sinonasal carcinomas (SNC) are a heterogeneous group of cancers that include keratinizing and non-keratinizing squamous cell carcinoma (SCC), spindle cell carcinoma, lymphoepithelial carcinoma, sinonasal undifferentiated carcinoma (SNUC), NUT carcinoma, neuroendocrine carcinomas (NEC), intestinal-type (ITAC), and non-intestinal-type adenocarcinoma (NITAC) (1). SNC represent most of the malignancies diagnosed in the sinonasal tract and their treatment is histology-driven (2–4). Thus, the reliable classification of SNC is paramount to guiding the treatment that the multi-disciplinary team will offer.

The current classification of SNC is mostly based on histomorphological features, in combination, when needed, with immunohistochemical and genetic studies. However, diagnosis of SNC is universally acknowledged as a challenge since several tumor types display overlapping features, and differential diagnosis includes a variety of entities. The fact that SNC exhibit some overlapping features from a morphological standpoint is not surprising, as several authors have demonstrated that the molecular features of these cancers are partially coinciding, and signatures of several genes are necessary to correctly classify diverse SNC (5, 6). The practical implications of this challenge are remarkable: not only is the sinonasal tract the site with the highest rate of major diagnostic discrepancy in the head and neck (19.0% vs. 0.0–8.3% in sinonasal and non-sinonasal sites, respectively) (7), but Choi et al. also demonstrated that initially misdiagnosed sinonasal cancers are associated with worse prognosis compared to those correctly identified prior to treatment (8). Moreover, even SCC diagnosis, which could be considered as relatively “simple” in most areas of the head and neck, has been associated with the highest rate of diagnostic discrepancy in the nasoethmoidal compartment (9). Correct classification of SNC is of paramount importance, particularly in the era of “histology-driven” management, as the best type and sequence of treatment modalities can significantly change with histology (2–4). As an example of that, neoadjuvant chemotherapy (ChT) has been adopted for several sinonasal cancers in an attempt to achieve a number of goals such as treatment intensification, chemoselection, orbit sparing, and reduction of distant failure. However, neoadjuvant ChT can display non-negligible toxicity, and no reliable means of response prediction are available. Thus, there exists a substantial uncertainty about the opportunity to start treatment with neoadjuvant ChT in some SNC.

These data dispel any doubt that the current method of classifying SNCs can be improved. Thus, research in the field of sinonasal oncology should be oriented toward the identification of novel clustering approaches to be implemented with the current classification. The main hypothesis of this study was that the reorganization of clinicopathological information on SNC could improve the prediction of prognosis and chemoradiosensitivity. The institutional series of SNC patients at the University of Brescia was reviewed and used to test the utility of machine learning techniques in exploiting commonly available information. The resulting experimental classifications were tested as prognostic and predictive factors and compared with the current means of classifying SNC.

## Materials and Methods

### Patients’ Selection and Data Acquisition

All patients affected by SNC who received curative treatment, including surgery, at the Unit of Otorhinolaryngology—Head and Neck Surgery of the University of Brescia between October 1998 and February 2019 were included in the analysis. ITAC and low-grade NITAC were excluded, as they were considered remarkably different clinical entities with respect to other SNCs (10).

The following information were retrospectively gathered for each case (full details are reported in **Table S1**): demographics, oncological history, treatment characteristics, response to neoadjuvant therapy (classified according to the Response Evaluation Criteria In Solid Tumors [RECIST], version 1.1), general pathologic features, cytomorphological information, histomorphological and local invasion-related information, (immuno)histochemical and nucleic acid-based test information, locoregional extension, follow-up events, and status at last evaluation. Differentiation of tumors was described through non-mutually exclusive classes (i.e., each tumor could be attributed to more than one differentiation class), based on the criteria summarized in **Table 1**. Margin status (11), perineural invasion (PNI) (11), lymphovascular invasion (LVI) (11), and infiltrative pattern-bone invasion (IPBI) (12) were considered as previously described. The 8th TNM Edition was employed (13).

**Table 1:** Summary of criteria to attribute squamous, glandular, neuroendocrine, mesenchymal, embryonal, and neural differentiation.

Differentiation	Attribution criteria*
Squamous	Squamous cytomorphology Keratinization Expression of p63 and/or p40
Glandular	Glandular cytomorphology Positive staining for periodic acid-Schiff stain, Alcian blue and/or mucicarmine Expression of cytokeratin 7, cytokeratin 20 and/or epithelial membrane antigen (MUC1/EMA)
Neuroendocrine	Expression of CD56, synaptophysin, chromogranin A and/or neuron-specific enolase (NSE)*
Mesenchymal	Presence of spindle cells, rhabdoid cells and/or osteoblastoid cells Expression of vimentin, muscle-specific actin, smooth muscle alfa-actin, calponin, myogenin, desmin and/or CD117
Embryonal	Expression of the carcinoembryonic antigen (CEA)
Neural	Expression of SOX10, NSE*, and/or glial fibrillary acid protein (GFAP)

*NSE was considered as a neural marker only when neuroendocrine markers were not expressed.

The pathologic evaluation of cases was led by the senior pathologist co-authoring this study (SB), who has 25 years of physician-level experience in the field, acquired in centers with a high volume of sinonasal cancers. A large majority of pathological reports (126/145, 86.9%) were either led or co-authored by SB. All non-SCC, non-conventional SCC, and nasoethmoidal SCC cases were analyzed in consensus by at least 2 dedicated head and neck pathologists and reviewed by SB.

### Unsupervised Re-Classification of Tumors

The softwares XLSTAT and RStudio were employed to perform the following analyses. The following experimental classifications were generated to test the main hypothesis of the study.

#### Unsupervised Re-Classification of Tumors Based on Pathological Features

Three groups of information (1—cytomorphological; 2—histomorphological and invasion-related; and 3—differentiation) underwent adjusted-inertia Multiple Correspondence Analysis (MCA). A minimum of 2 factors were extrapolated from each MCA, whereas the third or further factors were considered only if determining >10% of inertia. Agglomerative Hierarchical Clustering (AHC), which clusters observations through Euclidean dissimilarity as per Ward’s method, was applied to the factors extrapolated from MCAs. Three- to 6-cluster classifications were generated, and their association with disease-specific survival (DSS) was tested through the Cox proportional-hazards model. The classification providing the best prediction with minimum complexity was identified through analysis of the concordance index (C-index), Akaike Information Criterion (AIC), Bayesian Information Criterion (BIC), and Nagelkerke pseudo-R^2^ (NPR). This classification is hereby referred to as “classification #1.” The C-index expresses the goodness of fit of prognostic models. AIC and BIC estimate the prediction error of a model, whereas NPR determines how much of the variance observed in a series is explained by the variables (i.e., covariates). Thus, the higher the C-index and NPR, and the lower AIC and BIC, the better is one predictive model compared to another.

Each class of the selected classification was described in terms of cytomorphological, histomorphological- and invasion-related, and differentiation information through chi-square or Fisher’s exact test, as appropriate.

#### Unsupervised Re-Classification of Tumors Based on Differentiation Features

Differentiation information underwent AHC. Identification of the best classification in terms of the number of clusters was selected as described for classification #1. This classification is referred to as “classification #2.”

#### Unsupervised Re-Classification of Tumors Based on Locoregional Extension

Local and regional extension information was summarized through an adjusted-inertia MCA approach, as previously described, and underwent AHC. Identification of the best classification in terms of the number of clusters was selected with the same method described for classifications #1 and #2 but using local recurrence-free survival (LRFS) instead of DSS. This classification is referred to as “classification #3”.

### Prognostic Efficacy of Classifications #1 to #3 and Comparison With Available Classifications

The following time-to-event outcomes were considered to evaluate the prognostic efficacy of classifications: overall survival (OS), DSS, recurrence-free survival (RFS), LRFS, regional recurrence-free survival (RRFS), and distant recurrence-free survival (DRFS). The effect on prognosis was first tested through univariable analysis with a log-rank test (level of significance = 0.10). To measure a more reliable effect on outcomes, multivariable prognostic models were created through the Cox proportional-hazards method with an *a priori* selection of covariates for those outcomes which were impacted by classifications #1 to #3 at univariable analysis (level of significance = 0.05). The proportional hazards assumption was tested with Schoenfeld’s global test (level of significance = 0.05). Factors resulting in significant multivariable analysis for DSS, LRFS, and DRFS were also tested through a competing risk analysis with Gray’s test (level of significance = 0.05). Events were defined as appropriate to analyze competing risk for DSS (death of disease, death of other cause), LRFS (local recurrence, death without local recurrence), and DRFS (distant recurrence, death without distant recurrence). Causes of censorship were analyzed for DSS, RFS, LRFS, RRFS, and DRFS. Competing risk multivariable analysis was performed through a subdistribution hazard model for DSS, LRFS, and DRFS.

The following parameters were evaluated for classifications #1 to #3: independent prognostic effect (defined as the statistical significance of the classification when considered as a covariate in a multivariable model); prognostic segregation (defined as the ratio of observations clustered in a category significantly different from the reference category in a multivariable model out of the total number of patients in the series); and *a priori* applicability (evaluated as the confusion rate calculated through classification tree analysis run with the classification as the dependent variable and pathological or locoregional extension-related information as an explanatory variable, as appropriate).

Based on these parameters, classifications #1 and #2 were compared to the latest WHO classification of tumors, whereas classification #3 was compared to the pT category and stage of the latest TNM classification. Since the analysis was based on planned comparisons, multiple comparisons correction was not performed.

### Sub-Analysis of Patients Receiving Neoadjuvant Chemotherapy and Assessment of Chemoradiosensitivity

Descriptive statistics of the sub-cohort of patients receiving neoadjuvant ChT were performed. Multivariable models were applied to this sub-cohort of patients, including response to ChT as a covariate.

Chemoradiosensitivity (i.e., the tendency of the tumor to respond to ChT and/or radiotherapy (RT)) was estimated based on the criteria summarized in **Table 2**. The univariable association of response to neoadjuvant ChT and chemoradiosensitivity with demographics, oncological history, treatment characteristics, pathological information, and classifications #1 to #3 was tested with chi-square or Fisher’s exact test, as appropriate (level of significance = 0.10). A multivariable analysis of the same outcomes was performed using logistic regression applied to factors resulting significantly from the univariable analysis (level of significance = 0.05). The Classification Random Forest (CRF) method with “random with replacement” sampling, a subsample size of 50 observations, and the building of 100 classification trees was run to detect predictors of response to neoadjuvant ChT and chemoradiosensitivity.

**Table 2:** Summary of criteria to estimate chemoradiosensitivity of tumors.

Chemoradiosensitivity class	Criteria
**A**,“Highly chemoradiosensitive tumor”*	Complete response** following neoadjuvant ChT and/or curative-intended (ChT-)RT
**B**,“Moderately chemoradiosensitive tumor”*	Partial response** following neoadjuvant ChT At referral: local and/or regional relapse after a 2-year or longer disease-free interval since the date of completion of (ChT-)RT-including treatment During follow-up: local and/or regional relapse 2 or more years after completion of treatment including R1 surgery*** followed by adjuvant (ChT)-RT
**C**,“Chemoradioresistant tumor”*	Stable or progressing disease** after neoadjuvant ChT At referral: local and/or regional relapse within 2 years since the date of completion of (ChT-)RT-including treatment During follow-up: local and/or regional relapse within 2 years after completion of treatment including R1 surgery*** followed by adjuvant (ChT)-RT

*When a tumor had criteria designating multiple classes, the worst one was assigned. **Response was evaluated according to the Response Evaluation Criteria in Solid Tumors, version 1.1 (14). ***Patients receiving R0 surgery were excluded as chemoradiosensitivity could have been overestimated by completeness of resection.

## Results

### Cohort Description

The study included 145 patients, of whom 49 (33.8%) were women and 96 (66.2%) were men. Nine (6.2%) patients were treated between 1998 and 2000, 22 (15.2%) between 2001 and 2005, 30 (20.7%) between 2006 and 2010, 42 (29.0%) between 2011 and 2015, and 42 (29.0%) between 2016 and 2019 (**Figure S1**). The mean age at surgery was 63.8 years (median: 66.3; range: 28.8–89.0; interquartile range: 54.6–74.5).

Ninety-nine (68.3%) and 46 (31.7%) patients were referred for a primary or recurrent tumor, respectively. In the latter group, 15 (32.6%) patients had received surgery; 10 (21.7%) surgery and adjuvant RT; 7 (15.2%) surgery and adjuvant ChT-RT; 6 (13.0%) definitive ChT-RT, 3 (6.5%) RT; and 1 (2.2%) surgery and adjuvant ChT. In 4 (8.7%) of the patients, previous treatments could not be traced back. In patients referred after adjuvant or definitive (ChT-)RT (26/46, 56.5%), the disease-free interval was less than 1 year in 8 (30.8%) cases, between 12 and 24 months in 4 (15.4%), between 24 and 48 months in 4 (15.4%), between 5 and 10 years in 4 (15.4%), and beyond 10 years in 2 (7.7%).

Surgery consisted of endoscopic resection without transnasal craniectomy (ER), endoscopic resection with transnasal craniectomy (ERTC), cranioendoscopic resection (CER), open maxillectomy (OM), and endoscopic-assisted craniofacial resection (EA)CFR in 30 (20.7%), 21 (14.5%), 11 (7.6%), 48 (33.1%), and 35 (24.1%) patients, respectively. Neck dissection was performed in 30 (20.7%) patients, of whom 18 (60.0%) received a unilateral therapeutic comprehensive neck dissection and 12 (40.0%) unilateral superselective (I–IIA) or selective (I–III) neck dissection (when harvest of recipient vessels before microvascular reconstruction was indicated).

Fifty-six (38.6%) patients did not receive adjuvant treatments; 70 (48.3%) underwent adjuvant RT; 15 (10.3%) adjuvant RT-ChT; and 4 (2.8%) adjuvant ChT alone. Neoadjuvant ChT was administered to 35 (24.1%) patients, of whom 31 (88.6%) received docetaxel, cisplatin/carboplatin, 5-fluorouracile (TPF) regimen and 4 (11.4%) a cisplatin and etoposide alternated to adriamycin and ifosfamide (PE-AI) protocol.

### Pathological Features

**Figure 1:**
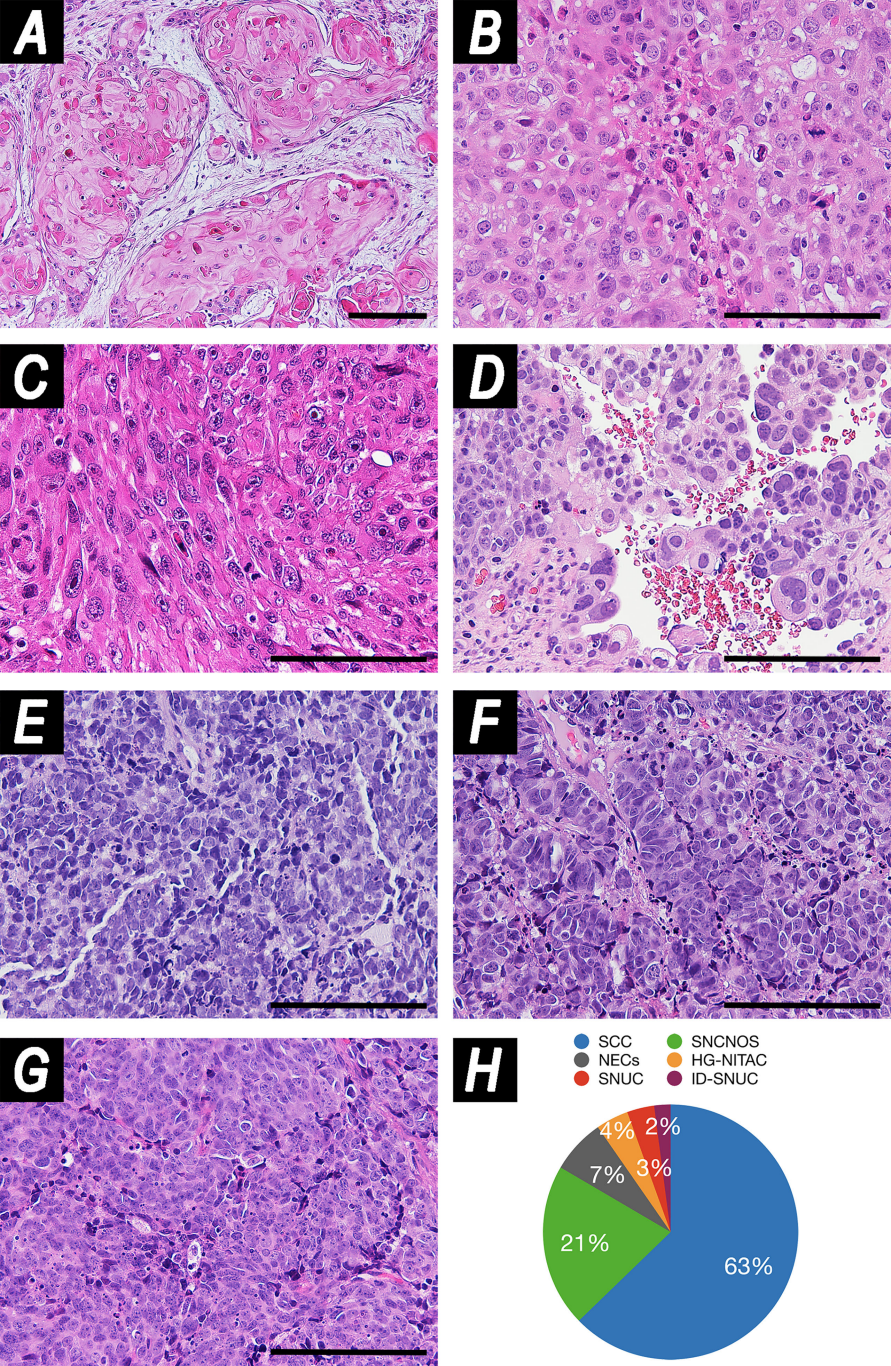
Panel illustrating examples of histologies included in the study. **(A)** Well-differentiated squamous cell carcinoma (SCC) (hematoxylin–eosin (HE), magnification: ×100). **(B)** Poorly differentiated SCC (HE, magnification: ×200). **(C)** Spindle cell carcinoma (HE, magnification: ×200). **(D)** High-grade non-intestinal-type adenocarcinoma (HG-NITAC) (HE, magnification: ×200). **(E)** Small cell neuroendocrine carcinoma (NEC) (HE, magnification: ×200). **(F)** Large cell NEC (HE, magnification: ×200). **(G)** Sinonasal undifferentiated carcinoma (SNUC) (HE, magnification: ×200). **(H)** Pie chart displaying distribution of histologies in the series. Scale bar: 100 μm. ID-SNUC, INI1/SMARCB1-deficient sinonasal undifferentiated carcinoma; SNCNOS, sinonasal carcinoma not otherwise specified.

The tumor epicenter was in the maxillary sinus and in the nasoethmoidal complex in 79 (54.5%) and 66 (45.5%) patients, respectively. Histology was distributed as follows: SCC in 91 (62.8%) patients (well/moderately differentiated in 31 [21.4%] cases, poorly differentiated in 60 [41.4%]), SNC not otherwise specified (SNCNOS) in 30 (20.7%), NEC in 10 (6.9%), high-grade NITAC (HG-NITAC) in 6 (4.1%), SNUC without molecular identifier in 5 (3.4%), and SMARCB1/INI1-deficient carcinoma (ID-SNUC) in 3 (2.1%) (**Figure 1**).

Of note, SNCNOS were poorly-to-non-differentiated SNCs that could not be classified as WHO-recognized entities. ID-SNUC was distinguished from SNUC owing to their substantially different clinical behavior (4). When considering SCCs, 64 (70.3%) were described as classical variants, 16 (17.6%) as non-keratinizing, 5 (5.5%) as adenosquamous, 3 (3.3%) as basaloid, 2 (2.2%) as spindle-cell, and 1 (1.1%) as adenomatoid. When considering NEC, 6 (60.0%) were described as small cells, 1 (10.0%) as large cells, and 3 (30.0%) were not otherwise specified. The preeminent grade of differentiation was described as low in 33 (22.8%) tumors, high in 93 (64.1%), and unspecified in 19 (13.1%). The worst grade of differentiation was low in 21 (14.5%) tumors, high in 105 (72.4%), and unpecified in 19 (13.1%). Inverted papilloma (IP) was found in 21 (14.5%) tumors, of which 19 (13.1%) were SCC and 2 (1.4%) SNCNOS. Margins were clear (R0) in 86 (59.3%) patients and involved (R+) in 59 (40.7%).

**Figure 2:**
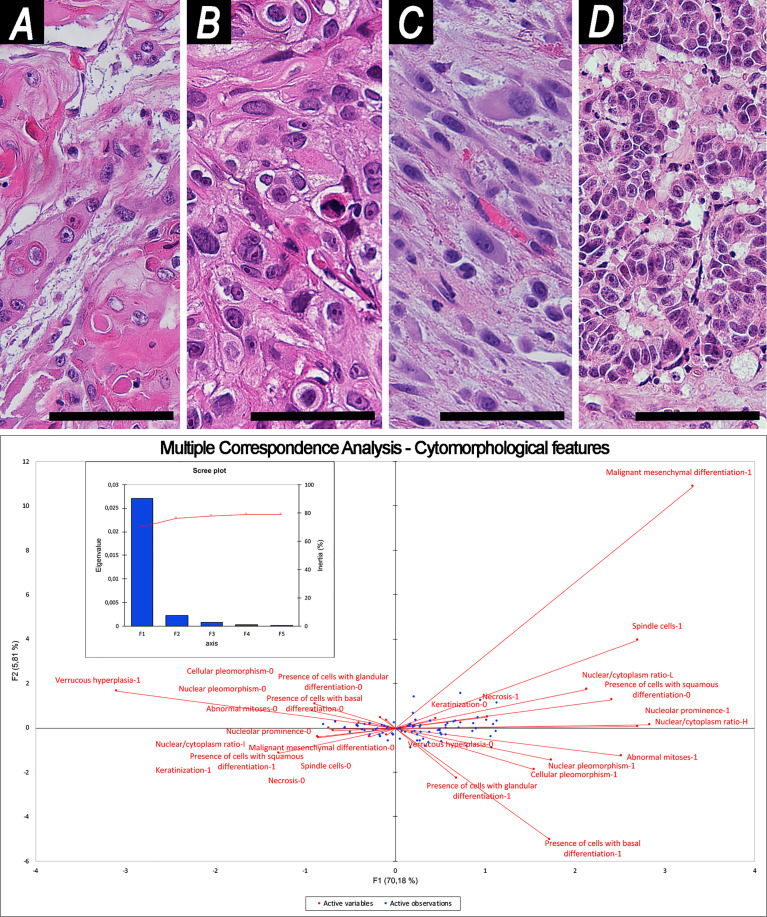
Examples of cytomorphology and related Multiple Correspondence Analysis. **(A, B)** Squamous cell morphology [**(A)** well-differentiated; **(B)** poorly-differentiated]. **(C)** Spindle cell morphology. **(D)** Glandular cell morphology. Magnification of histological images is ×200; all are stained through hematoxylin–eosin. The bottom image shows organization of variables into cartesian axes depending on their mutual relationships. This results in 2 factors (F1, F2), represented in the y- and x-axes of the graph, which reliably summarize sample variability, as shown in the scree plot. Scale bar: 50 μm.

Squamous morphology of tumor cells was observed in 107 (73.8%) cases, basaloid in 12 (8.3%), glandular in 18 (12.4%), and mesenchymal in 18 (12.4%), out of which 14 (9.7%) were spindle, 3 (2.1%) rhabdoid, and 1 (0.7%) osteoblastoid (**Figure 2**).

Keratinization was found in 38 (26.2%) cases, cellular pleomorphism in 53 (36.6%), nuclear pleomorphism in 50 (34.5%), nucleolar prominence in 30 (20.7%), abnormal mitoses in 20 (13.8%), neoplastic necrosis in 66 (45.5%), and verrucous hyperplasia in 3 (2.1%). The nucleus-to-cytoplasm ratio was classified as high in 22 (15.2%) tumors, low in 9 (6.2%), and intermediate or unspecified in 114 (78.6%).

**Figure 3:**
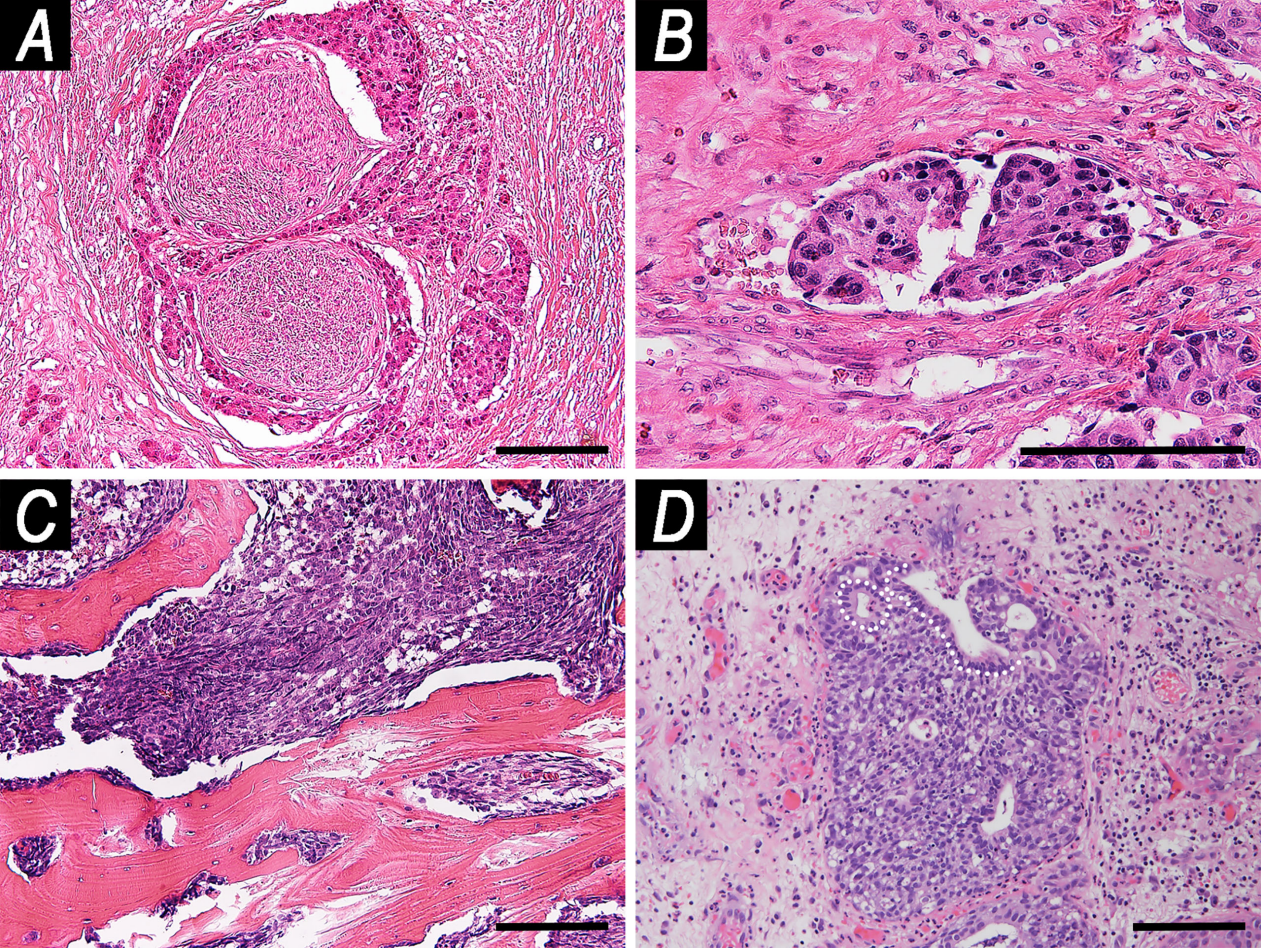
Examples of microscopic local spread patterns. **(A)** Perineural invasion (hematoxylin–eosin (HE), magnification: ×100). **(B)** Endovascular tumor embolization (HE, magnification: ×200). **(C)** Infiltrative pattern-bone invasion (HE, magnification: ×100). **(D)** Pagetoid growth (HE, magnification: ×100). White dashed line indicates the basal lamina of glandular epithelium of a submucosal gland. The tumor grew along the glandular axis underneath the epithelium. Scale bar: 100 μm.

Pattern of growth was described as solid in 125 (86.2%) tumors, papillary in 27 (18.6%), transitional-like in 8 (5.5%), lobular in 7 (4.8%), cribriform in 7 (4.8%), pagetoid in 7 (4.8%), and tubular in 2 (1.4%). Overall, PNI was observed in 44 (30.3%) cancers, and LVI in 49 (33.8%). Infiltrative-type bone invasion was observed in 85 (58.6%) patients (**Figure 3**).

According to the criteria reported in **Table 1**, squamous differentiation was observed in 114 (78.6%) tumors, glandular in 35 (24.1%), mesenchymal in 32 (22.1%), neuroendocrine in 25 (17.2%), neural in 3 (2.1%), and embryonic in 1 (0.7%). Given their rarity in the series, neural and embryonic differentiations were not considered further.

Histochemical, (immuno)histochemical, and nucleic acid-based staining employed over the study period are reported in **Table S2**. Epstein–Barr virus (EBV), human papilloma virus (HPV), and polyomavirus were searched in 16 (11.0%), 3 (2.1%), and 3 (2.1%) cases, respectively. Only one tumor was found to be EBV+. No cases associated with HPV or polyomavirus were observed. A positive stain for p16 was found in 5 of 9 (55.6%) cases in which it was tested.

The tumor involved the orbital content in 41 (28.3%) cases, the bony skull base in 41 (28.3%), the dura mater in 26 (17.9%), masticator and/or parapharyngeal space in 46 (31.7%), the facial soft tissues in 46 (31.7%), the sphenoid sinus in 37 (25.5%), the frontal sinus in 19 (13.1%), and the nasopharynx in 24 (16.6%). The pathological T category was distributed as follows: pT1 in 12 (8.3%) patients, pT2 in 16 (11.0%), pT3 in 22 (15.2%), pT4a in 43 (29.7%), and pT4b in 52 (35.9%). Eighteen (12.4%) patients had pathologically proven nodal metastases. The tumor stage was classified as I in 12 (8.3%) patients, II in 15 (10.3%), III in 21 (14.5%), IVA in 42 (29.0%), and IVB in 55 (37.9%).

### Oncologic Outcomes

The mean follow-up duration was 48.2 months (median: 29.7; range: 0.8–215.6; inter-quartile range: 9.4–73.2). The status of patients at last contact was distributed as follows: died of disease in 61 (42.1%) patients; alive with no evidence of disease in 60 (41.4%); died of other causes in 7 (4.8%); and alive with evidence of disease in 4 (2.8%). Thirteen (9.0%) patients were lost at follow-up. The following data refer to the subgroup of patients for whom follow-up information is available (n = 132).

**Figure 4:**
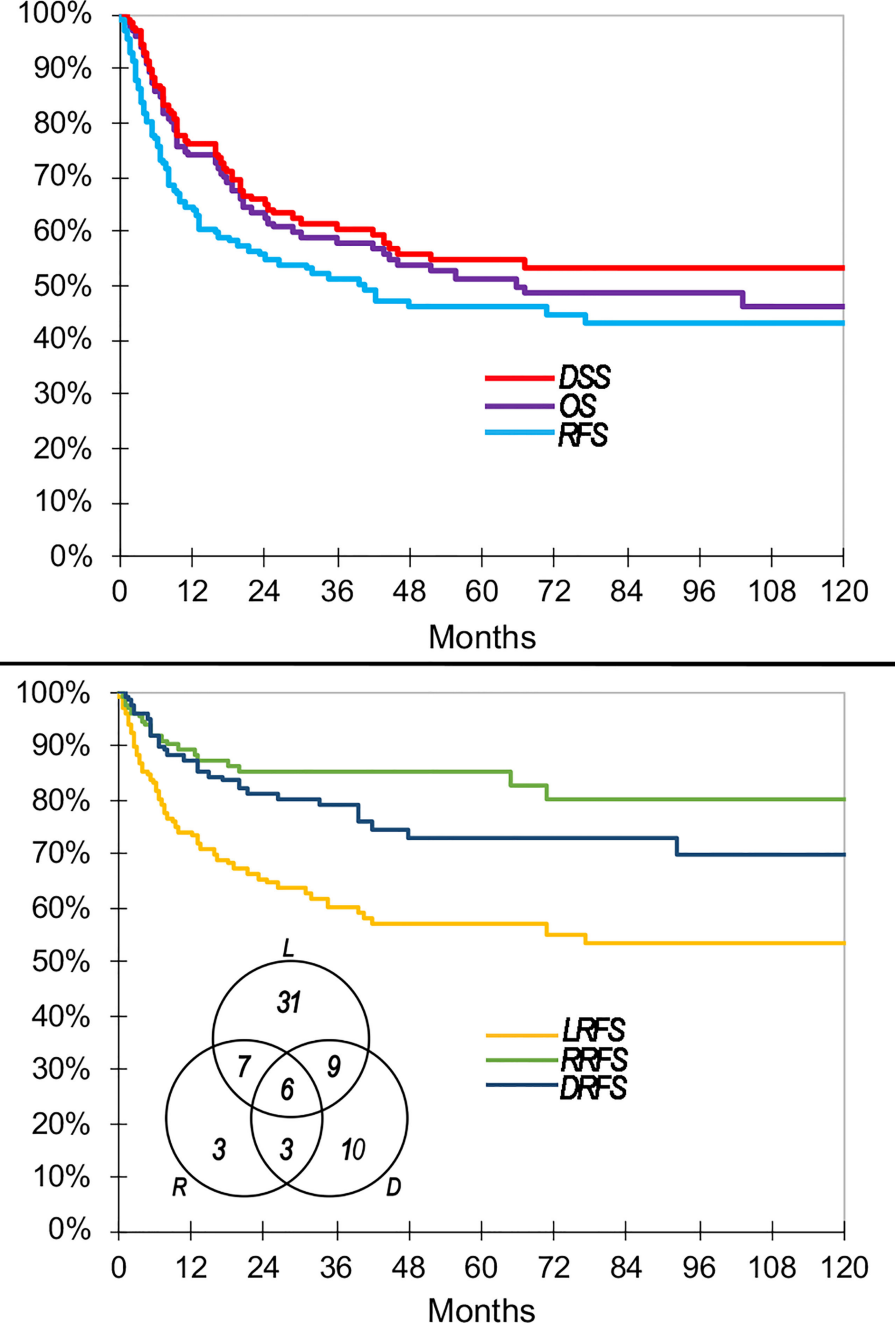
Main oncologic outcomes of the series summarized through Kaplan–Meier curves. Venn diagram shows raw count of recurrences. DRFS, distant recurrence-free survival; DSS, disease-specific survival; LRFS, local recurrence-free survival; OS, overall survival; RFS, recurrence-free survival; RRFS, regional recurrence-free survival.

One-, 2-, 5-, and 10-year OS were 74.0, 62.5, 51.3, and 46.3%, respectively (**Figure 4**); 90% of deaths from any cause occurred within 55 months after diagnosis. One-, 2-, 5-, and 10-year DSS were 76.0, 65.0, 54.6, and 53.1%, respectively (**Figure 4**); 90% of cancer-specific deaths occurred within 50 months after diagnosis.

Sixty-nine (52.3%) patients had at least 1 recurrence. In particular, there were 31 (23.5%) local recurrences, 7 (5.3%) locoregional, 9 (6.8%) local and distant, 3 (2.3%) regional, 3 (2.3%) regional and distant, 10 (7.6%) distant, and 6 (4.5%) locoregional and distant (**Figure 4**). Cumulatively, recurrence was observed at the local, regional, and distant sites in 53 (40.2%), 19 (14.4%), and 28 (21.2%) patients, respectively.

One-, 2-, 5-, and 10-year RFS were 64.6, 54.9, 46.1, and 43.0%, respectively (**Figure 4**). One-, 2-, 5-, and 10-year LRFS were 74.3, 65.5, 57.0, and 53.3%, respectively (**Figure 4**). One-, 2-, 5-, and 10-year RRFS were 89.3, 85.1, 85.1, and 80.2%, respectively (**Figure 4**). One-, 2-, 5-, and 10-year DRFS were 87.4, 81.4, 73.1, and 70.1%, respectively (**Figure 4**). The time to observe 90% of any recurrence, local recurrence, regional recurrence, and distant recurrence was 33, 35, 32, and 43 months, respectively. The causes of censorship in the RFS (n = 63) analysis were distributed as follows: 6 (9.5%) patients died of other causes with no recurrence of disease, and 57 (90.5%) patients were alive with no recurrence of disease. The causes of censorship in the LRFS (n = 79) analysis were distributed as follows: 6 (7.6%) patients died of other causes with no local recurrence of disease, 15 (19.0%) patients died of non-locally recurrent disease, and 58 (73.4%) patients were alive with no local recurrence of disease. The causes of censorship in the RRFS (n = 113) analysis were distributed as follows: 6 (5.3%) patients died of other causes with no regional recurrence of disease, 43 (38.1%) patients died of non-regionally recurrent disease, 64 (56.6%) patients were alive with no regional recurrence of disease. The causes of censorship in the DRFS (n = 104) analysis were distributed as follows: 7 (6.7%) patients died of other causes with no distant recurrence of disease, 32 (30.8%) patients died of recurrent disease without distant metastasis, and 65 (62.5%) patients were alive with no distant recurrence of disease.

A multivariable analysis of prognostic factors is reported in **Tables S3**–**S6**. Schoenfeld’s global p-value was >0.05 for all models. The results of the competing risk analysis of DSS and LRFS are summarized in **Tables S4**, **S6**, respectively. Gray’s test competing risk analysis of DRFS showed that both covariates showing significance at multivariable analysis (i.e., histology according to WHO classification and locoregional extension according to classification #3) are potentially associated with informative censoring bias. The probability of informative censorship is significantly affected by the category of covariates (p = 0.027 and p <0.0001, respectively). While the locoregional extension maintained significance in terms of distant recurrence-specific events (p = 0.008), histology lost significance (p = 0.408). The multi-variable subdistribution hazard models of DSS and LRFS did not show relevant difference compared with the respective Cox proportional hazards models, thus excluding the presence of a relevant informative censoring bias (**Table S7**). Multivariable subdistribution hazard models of DRFS showed a relevant difference compared with the respective Cox proportional hazards models (i.e., the covariate histology lost significance) (**Table S7**). Thus, the DRFS Cox proportional hazard model was considered flawed by informative censoring and was not used to compare classifications. Given the paucity of regional failure events (n = 19) and the remarkable number of competing events, RRFS was also excluded from terms of comparison of classifications.

### Classifications #1 and #2 and Comparison With WHO Classification

Cytomorphological-MCA, histomorphological and invasion-related-MCA, and differentiation-MCA generated 2 factors each, representing 76.0, 70.7, and 82.4% of variability in observations, respectively.

For classification #1, 5- and 6-cluster classifications were the best combinations of AIC/BIC and NPR (**Table S8**); since the 6-cluster classification had a 1-case class, the 5-cluster classification was selected due to better sorting of cases. Classification #1 is reported in **Table 3**. An independent prognostic effect was observed on LRFS and prognostic segregation was 15.9% (“NEC with mesenchymal features” vs. others). *A priori* applicability was suboptimal, with a confusion rate of 4.1%.

**Table 3:** Classification #1 and class-specific outcomes.

Classification #1	Class 1 (n = 65)	Class 2 (n = 23)	Class 3 (n = 18)	Class 4 (n = 16)	Class 5 (n = 23)
**Brief description**	“Squamous cell carcinoma”	“Spindle cell and adenosquamous carcinoma”	“Papillary squamous cell carcinoma, possibly ex-inverted papilloma”	“Neuroendocrine carcinomas with glandular features”	“Neuroendocrine carcinomas with mesenchymal features”
**Cytomorphological features**	Squamous morphology (98.5%) Keratinization (43.1%) Rare cellular (16.9%) and nuclear (20.0%) pleomorphism Intermediate nucleus/cytoplasm ratio (95.4%) Variable preeminent grade (G1/2: 40.0%; G3/4/X: 60.0%)	Squamous morphology (87.0%) Keratinization (26.1%) Glandular morphology (39.1%) Spindle cell (34.8%) or other mesenchymal morphology (4.3%) Frequent cellular (60.9%) and nuclear (47.8%) pleomorphism Nucleolar prominence (26.1%) Intermediate nucleus/cytoplasm ratio (95.4%) High-grade (100.0%)	Squamous morphology (100.0%) Keratinization (22.2%) Possible cellular (38.9%) and nuclear (38.9%) pleomorphism Intermediate nucleus/cytoplasm ratio (88.9%) Variable preeminent grade (G1/2: 27.8%; G3/4/X: 72.2%)	Rare squamous morphology (18.8%) Frequent cellular (62.5%) and nuclear (50.0%) pleomorphism Nucleolar prominence (43.8%) High nucleus/cytoplasm ratio (56.3%) High-grade (100.0%)	Rare squamous morphology (8.7%) Glandular morphology (39.1%) Spindle cell (26.1%) or other mesenchymal morphology (13.0%) Frequent cellular (47.8%) and nuclear (47.8%) pleomorphism Nucleolar prominence (52.2%) High (34.8%) or low (21.7%) nucleus/cytoplasm ratio High-grade (91.3%)
**Histomorphological features**	Solid architecture (98.5%) Association with inverted papilloma (17.2%)	Solid architecture (91.3%) Cribriform architecture (17.4%) Association with inverted papilloma (13.0%)	Solid architecture (22.2%) Papillary architecture (83.3%) Association with inverted papilloma (38.9%)	Solid architecture (93.8%)	Solid architecture (91.3%) Cribriform architecture (13.0%)
**Invasion-related features**	PNI (38.5%) LVI (46.2%) IPBI (69.2%)	PNI (47.8%) LVI (30.4%) IPBI (56.5%)	PNI (0.0%) LVI (0.0%) IPBI (16.7%)	PNI (6.3%) LVI (31.3%) IPBI (75.0%)	PNI (30.4%) LVI (30.4%) IPBI (52.2%)
**Differentiation features**	Squamous (100.0%)	Squamous (100,0%) Glandular (73.9%) Mesenchymal (65.2%)	Squamous (100.0%)	Glandular (78.3%) Neuroendocrine (75.0%) Squamous (25.0%)	Mesenchymal (73.9%) Neuroendocrine (50.0%) Squamous (17.4%)
Multivariable model-adjusted* impact on LRFS (HR (95%-CI), p-value)	REF	1.83 (0.84–3.99), p = 0.130	0.21 (0.03–1.72), p = 0.144	1.18 (0.26–5.22), p = 0.833	3.47 (1.32–9.13), **p = 0.012**
Multivariable model-adjusted** impact on RRFS (HR (95%-CI), p-value)	REF	0.69 (0.14–3.52), p = 0.658	N.A., p = 0.998	2.66 (0.41–17.24), p = 0.305	8.66 (2.49–30.06), **p = 0.001**

95% CI, 95% confidence interval; G1, well differentiated; G2, moderately differentiated; G3, poorly differentiated; G4, undifferentiated; GX, grade of differentiation not specified or not assessable (lesions defined as “high-grade” regardless of pathological features); HR, hazard ratio; IPBI, infiltrative pattern-bone invasion; LVI, lymphovascular invasion; N.A., not assessable; PNI, perineural invasion; REF, reference. *Multivariable model included: classification #1, type of surgery, classification # 3, margin status, adjuvant treatment, previous chemotherapy. **Multivariable model included: classification #1, neck dissection, orbital involvement, involvement of the masticator and/or parapharyngeal space, facial tissues involvement, sphenoid sinus involvement, margin status, adjuvant treatment. P-values less than 0.05 are highlighted in bold.

**Figure 5:**
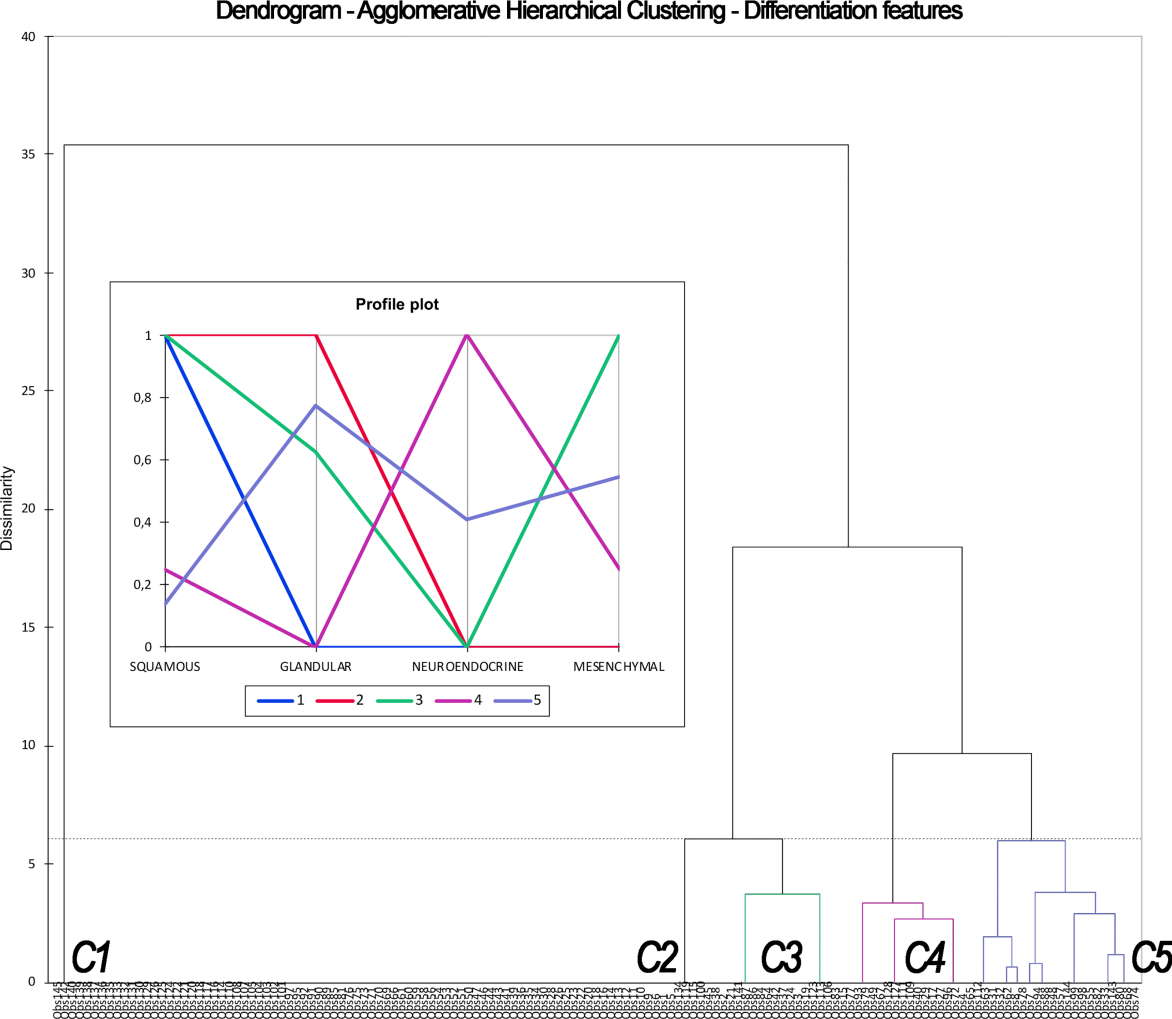
Dendrogram and profile plot summarizing the process of Agglomerative Hierarchical Clustering based on differentiation features (i.e., leading to classification #2). This unsupervised machine learning methodology clusters observations (listed along the x-axis) based on their dissimilarity (expressed in the y-axis). Dissimilarity, which is defined according to differentiation, is maximal between clusters and minimal within each cluster. The process resulted in 5 clusters (C1–5), each one displaying a determinate frequency of squamous, glandular, neuroendocrine, and mesenchymal differentiation, as expressed by the profile plot. C1, C2, C3, C4, and C5 corresponds to cluster labeled as “squamous cell carcinoma,” “squamous cell carcinoma with glandular features,” “squamous cell carcinoma with mesenchymal features,” “neuroendocrine carcinomas without glandular features,” and “other carcinomas” in **Table 4**.

**Figure 6:**
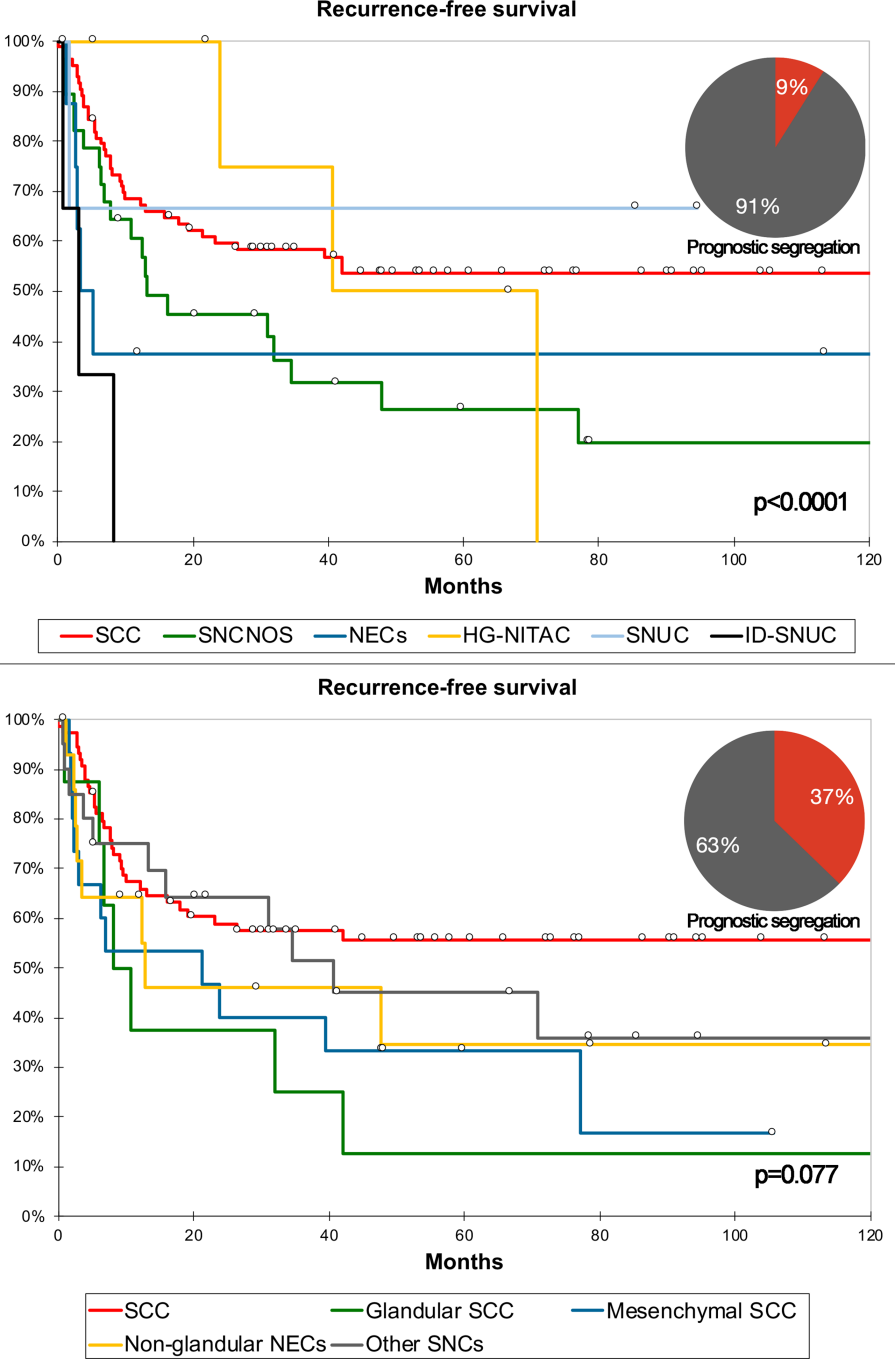
Kaplan–Meier curves depicting recurrence-free survival of different sinonasal carcinomas (SNC) classified according to the WHO criteria and classification #2. Prognostic segregation is expressed through pie charts. P-value refers to log-rank test (see **Tables S5** and **4** for multivariable-adjusted significance). See **Table 4** for detailed definition of each group of carcinomas as per classification #2. HG-NITAC, high-grade non-intestinal-type adenocarcinoma; ID-SNUC, INI1-SMARCB1-deficient sinonasal undifferentiated carcinoma; NEC, neuroendocrine carcinoma; SCC, squamous cell carcinoma; SNCNOS, sinonasal carcinoma not otherwise specified; SNUC, sinonasal undifferentiated carcinoma.

**Figure 7:**
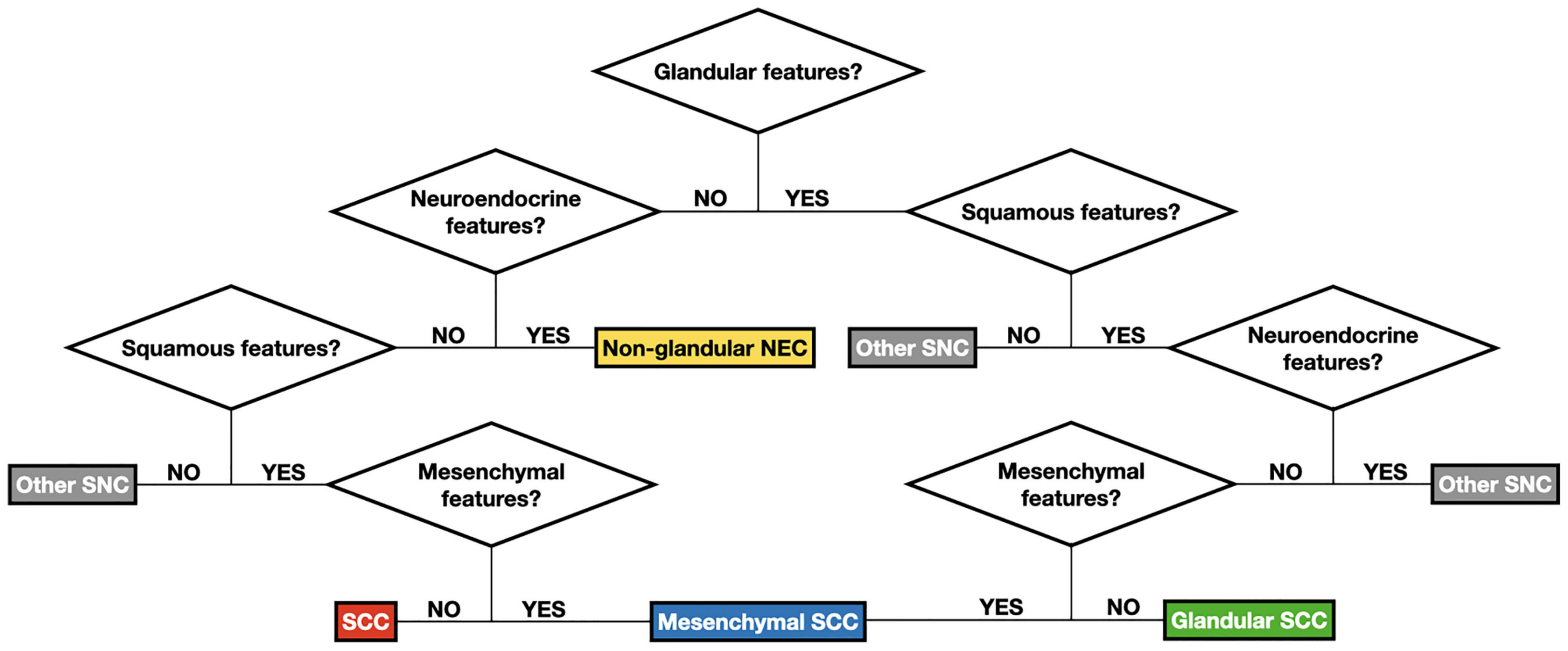
Flow chart summarizing the logical steps to classify sinonasal carcinomas (SNC) according to classification #2. See **Table 1** for detailed description of features designating the differentiation(s) of carcinomas. See **Table 4** for de-tailed definition of each group of carcinomas as per classification #2. NEC, neuroendocrine carcinoma; SCC, squamous cell carcinoma.

For classification #2, the 5-cluster classification generated through AHC (**Figure 5**) was associated with the best combination of AIC/BIC and NPR (**Table S9**). Classification #2 is reported in **Table 4**. An independent prognostic effect was observed on RFS and LRFS. Prognostic segregation was 37.2% for RFS (“SCC with mesenchymal features”, “NEC without glandular features”, and “other carcinomas” vs. others) and 26.2% for LRFS (“SCC with mesenchymal features” and “other carcinomas” vs. others) (**Figure 6**). The *a priori* applicability was optimal, with a confusion rate of 0.0% (**Figure 7**). The WHO classification (i.e., classification in SCC, SNCNOS, NEC, HG-NITAC, SNUC, and ID-SNUC) had an independent prognostic effect on OS, DSS, RFS, and DRFS. Prognostic segregation was 9.0% (NEC and ID-SNUC vs. others).

**Table 4:** Classification #2 and class-specific outcomes.

Classification #2	Class 1 (n = 83)	Class 2 (n = 8)	Class 3 (n = 16)	Class 4 (n = 16)	Class 5 (n = 22)
**Brief description**	“Squamous cell carcinoma”	“Squamous cell carcinoma with glandular features”	“Squamous cell carcinoma with mesenchymal features”	“Neuroendocrine carcinomas without glandular features”	“Other carcinomas”
**Label in** **Figure 5**	“C1”	“C2”	“C3”	“C4”	“C5”
**Label in** **Figures 6** **and** **7**	“SCC”	“Glandular SCC”	“Mesenchymal SCC”	“Non-glandular NEC”	“Other SNC”
**Differentiation**	Squamous (100.0%) Glandular (0.0%) Mesenchymal (0.0%) Neuroendocrine (0.0%)	Squamous (100.0%) Glandular (100.0%) Mesenchymal (0.0%) Neuroendocrine (0.0%)	Squamous (100.0%) Glandular (62.5%) Mesenchymal (100.0%) Neuroendocrine (0.0%)	Squamous (25.0%) Glandular (0.0%) Mesenchymal (25.0%) Neuroendocrine (100.0%)	Squamous (13.6%) Glandular (77.3%) Mesenchymal (54.5%) Neuroendocrine (40.9%)
Multivariable model-adjusted* impact on RFS (HR (95%-CI), p-value)	REF	2.07 (0.84–5.09), p = 0.112	2.42 (1.10–5.34), **p = 0.028**	8.50 (2.60–27.74), **p = 0.0004**	2.32 (1.03–5.23), **p = 0.043**
Multivariable model-adjusted** impact on LRFS (HR (95%-CI), p-value)	REF	1.67 (0.57–4.88), p = 0.352	2.96 (1.25–7.03), **p = 0.014**	4.00 (0.86–18.59), p = 0.078	3.59 (1.42–9.08), **p = 0.007**

95% CI, 95% confidence interval; HR, hazard ratio, *Multivariable model included: classification #2, type of surgery, classification # 3, margin status, adjuvant treatment. **Multivariable model included: classification #2, type of surgery, classification # 3, margin status, adjuvant treatment, previous chemotherapy. P-values less than 0.05 are highlighted in bold.

When comparing RFS multivariable models including the type of surgery, locoregional extensions summarized as classification #3, margin status, type of adjuvant treatment, and either classification #2 or WHO classification, C-index, AIC, BIC, and NPR were 0.484, 577, 613, and 0.774 vs. 0.431, 579, 617, and 0.781, respectively (**Table 5**).

**Table 5:** Comparison of multivariable models in terms of concordance index (C-index), Akaike Information Criterion (AIC), Bayesian Information Criterion (BIC), and Nagelkerke pseudo-R^2^ (NPR).

Model	C-index	AIC	BIC	NPR
Classification #2—RFS*	0.484	577	613	0.774
WHO—RFS*	0.431	579	617	0.781
Classification #3—OS**	0.598	529	566	0.773
Pathological T category (8th ed.)—OS**	0.318	540	577	0.717
Tumor stage (8th ed.)—OS**	0.312	545	582	0.725
Classification #3—DSS**	0.608	467	502	0.794
Pathological T category (8th ed.)—DSS**	0.322	478	513	0.748
Classification #3—RFS**	0.431	579	617	0.781
Pathological T category (8th ed.)—RFS**	0.306	591	629	0.757
Tumor stage (8th ed.)—RFS**	0.321	595	633	0.719
Classification #3—LRFS**	0.576	452	460	0.422
Pathological T category (8th ed.)—LRFS**	0.353	460	470	0.387
Tumor stage (8th ed.)—LRFS**	0.348	460	468	0.362

DSS, disease-specific survival; LRFS, local recurrence-free survival; OS, overall survival; RFS, recurrence-free survival; WHO, World Health Organization. *Model included type of surgery, locoregional extensions summarized as classification #3, margin status, type of adjuvant treatment, and either classification #2 or WHO classification of histology. **Model included WHO classification of histology, type of surgery, margin status, type of adjuvant treatment, and locoregional extensions summarized as either classification #3, pathological T category or tumor stage.

### Classification #3 and Comparison With TNM Classification

Locoregional extension-related MCA generated 2 factors, representing 74.1% of the variability in observations. Five-cluster classification was associated with the best combination of AIC/BIC and NPR (**Table S10**). Classification #3 is reported in **Table 6**. An independent prognostic effect was observed on OS, DSS, RFS, and LRFS. Prognostic segregation was 33.8% for OS, DSS, RFS, and LRFS (“transcranial,” “spheno-infracranial,” and “fronto-orbito-basal” vs. others). *A priori* applicability was suboptimal, with a confusion rate of 3.4%.

**Table 6:** Classification #3 and class-specific outcomes.

Classification #3	Class 1 (n = 64)	Class 2 (n = 32)	Class 3 (n = 18)	Class 4 (n = 14)	Class 5 (n = 17)	P-value
**Brief description**	“Sinonasal”	“Facial”	“Transcranial”	“Spheno-infracranial”	“Fronto-orbito-basal”	–
**Orbital infiltration**	3.1%	46.9%	7.1%	44.4%	88.2%	<0.0001
**Infiltration of the bony skull base**	10.9%	9.4%	100.0%	22.2%	76.5%	<0.0001
**Dural infiltration**	0.0%	0.0%	100.0%	0.0%	70.6%	<0.0001
**Infiltration of the masticator/parapharyngeal space**	17.2%	50.0%	0.0%	83.3%	23.5%	<0.0001
**Infiltration of facial tissues**	0.0%	96.9%	0.0%	38.9%	47.1%	<0.0001
**Infiltration of sphenoid sinus**	6.3%	12.5%	35.7%	77.8%	58.8%	<0.0001
**Infiltration of frontal sinus**	6.3%	0.0%	0.0%	0.0%	88.2%	<0.0001
**Nasopharyngeal infiltration**	6.3%	0.0%	0.0%	88.9%	23.5%	<0.0001
**Nodal metastasis**	9.4%	15.6%	0.0%	16.7%	23.5%	0.256
**5-year LRFS (95% CI)**	80.2% (71.4–89.0%)	57.1% (36.0–78.1%)	33.3% (3.4–63.3%)	22.0% (0.0–46.1%)	13.3% (0.00–34.9%)	<0.0001

95% CI, 95% confidence interval; LRFS, local recurrence-free survival. Multivariable model included: classification #3, type of surgery, classification #2, margin status, adjuvant treatment. Multivariable model included: classification #3, type of surgery, classification#2, margin status, adjuvant treatment, previous chemotherapy.

Pathological T category (i.e., classification in pT1, pT2, pT3, pT4a, and pT4b) had an independent prognostic effect on OS, DSS, RFS, and LRFS. Prognostic segregation was 35.9% (pT4b vs. others) for OS, DSS, and RFS, and 65.5% for LRFS (pT4a and pT4b vs. others). The tumor stage (i.e., classification into stage I, II, III, IVA, and IVB) had an independent prognostic effect on OS, RFS, and LRFS. Prognostic segregation was 37.9% (IVB vs. others) for OS and RFS, and 66.9% for LRFS (IVA and IVB vs. others).

### Response to Neoadjuvant Chemotherapy and Chemoradiosensitivity

Response to neoadjuvant ChT was distributed as follows: partial response (PR) in 15/35 (42.9%) patients; stable disease (SD) in 15/35 (42.9%); and progression of disease (PD) in 5/35 (14.3%). Histology was SCC in 14/35 (40.0%) patients, SNCNOS in 11/35 (31.4%), SNEC in 4/35 (11.4%), and SNUC, HG-NITAC, and ID-SNUC in 2/35 (5.7%) each. Fourteen/35 (40.0%) tumors were classified as pT4b, 12/35 (34.3%) as pT4a, 7/35 (20.0%) as pT3, and 2/35 (5.7%) as pT2. Chemoradiosensitivity could be estimated in 76/145 (52.4%) patients and was distributed as follows: class A in 2 (2.6%) cases, class B in 33 (43.4%), and class C in 41 (53.9%).

Among all the tested information, only PNI and pagetoid growth were significantly associated with the response to ChT (p = 0.043 and p = 0.070, respectively, with PNI being associated with lower rate of PR and higher rate of PD, and pagetoid growth with higher rate of PR), neither of which maintained significance at multivariable analysis. The estimate of chemoradiosensitivity was associated with cellular pleomorphism (p = 0.030), solid pattern of growth (p = 0.056), pagetoid growth (p = 0.032), PNI (p = 0.001), and classification #1 (p = 0.050). However, only pagetoid growth and PNI-maintained significance at logistic regression (p = 0.030 and p = 0.007, respectively), with pagetoid growth being significantly associated with class A or B and PNI with class C. CRF was associated with a steady out-of-the-basket error between 40 and 60% when applied to both the response to neoadjuvant ChT and chemoradiosensitivity.

When the prognosis of patients receiving neoadjuvant ChT was analyzed in the multivariable model, the response to ChT affected OS, DSS, and RFS, with PD being associated with a significantly worse outcome. While margin status played a substantial prognostic role in multivariable models for all outcomes but RRFS and DRFS, its prognostic effect was lost when the analysis was limited to the subset of patients treated with neoadjuvant ChT.

## Discussion

### Heterogeneity of Sinonasal Carcinomas

The first significant confirmatory finding of this study is that cancers grouped under the term “SNC” are extremely heterogeneous. While some dominant features, such as squamous cell morphology and a solid pattern of growth, could be demonstrated, numerous tumors displayed diverse pathological features from cytomorphological, histomorphological, and immunohistochemical standpoints. As already highlighted by other authors (15–17), this emphasizes that carcinomas of the sinonasal tract definitely have overlapping features, which probably explains difficulties in diagnosis, the high rate of diagnostic discrepancies, and suboptimal prediction of treatment response. As a glaring example of this phenomenon, according to classification #2, 62/145 (42.8%) of cases were classified as non-purely squamous SNC, of which, however, 31 (50.0%) displayed at least one of the features required to be labeled as “squamous.”

### Steadily Poor Prognosis of Sinonasal Carcinomas

Another relevant result of our analysis is that a substantial proportion of SNCs were associated with poor prognosis even if treatment was performed over the last 2 decades within a modern, multidisciplinary frame. As reported by Dulguerov et al., the OS of SNC progressively increased in the second half of the last century, from 28% ± 13% in the 1960s, to 36% ± 13% in the 1970s, 43% ± 15% in the 1980s, and 51% ± 14% in the 1990s (18). According to our results and consistent with a Danish population-based phase-4 cohort study performed in 2008–2015 (19), the positive trend observed by Dulguerov et al. has plateaued, with 5-year OS settled roughly around 50%. Of note, around one-third of patients included in the present series died within one year from the end of treatment, mostly owing to an early local recurrence, highlighting that a remarkable proportion of SNC is highly aggressive and poorly controlled, even if they were initially considered eligible for curative treatment. On one hand, this might be related to the large number of patients with locally advanced SNC in our study (T3/4: 117/145, 80.7%; vs. 61.4% in the recently published Danish Head and Neck Cancer (DAHANCA) group study) (19). On the other hand, this finding suggests that a relevant subgroup of SNC is not managed effectively even with contemporary treatment strategies. Interestingly, SNCNOS, namely SNC lacking a precise diagnosis according to the WHO criteria, were associated with dismal OS and DSS similarly to NEC and ID-SNUC (5-year estimates: 36.6 and 41.7%, 33.3 and 37.5%, and 0.0 and 0.0%, respectively). SNUCs with normal or non-tested expression of SMARCB1/INI1, were associated with 5-year OS and DSS of 66.7%, which aligns with the 59% rate recently reported by Amit et al. in a cohort of 95 SNUCs (20). While SNUC is still considered as a wastebasket entity, the progressive exclusion from this category of aggressive SNC with specific molecular identifiers such as ID-SNUC (21), SMARCA4-deficient carcinoma (22) and NUT carcinoma (23), together with the increasing use of neoadjuvant ChT-based regimens, has led to considerable improvement in SNUC-specific outcomes, particularly when treatment is based on chemoselection (20). Our data suggest that poorly understood SNC currently bears a worse prognosis than SNUC, which in the past was unanimously considered to be associated with a dismal outcome (24–26). Among SNC with the worst prognosis, NEC and ID-SNUC represented only a small proportion (13/145, 9.0%), while SNCNOS was the second most frequent diagnosis after SCC, with 30 (20.7%) cases. This further emphasizes the difficulty of reaching a WHO-recognized SNC diagnosis in SNC and the need for better classification of SNC to improve treatment outcomes.

### The Impact of Multimodal Treatment

**Figure 8:**
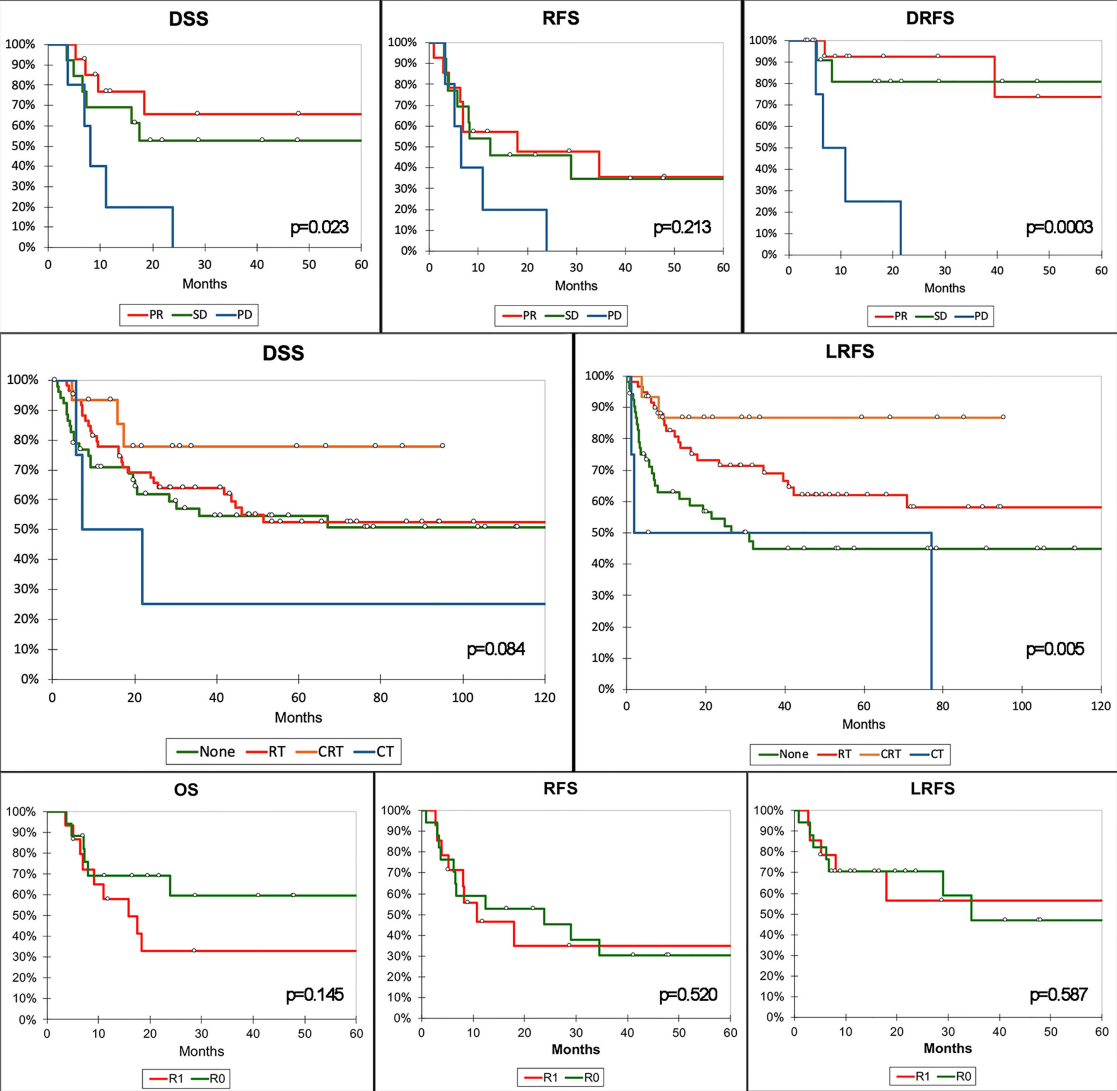
Kaplan–Meier curves summarizing the most relevant results of the survival analysis. Top row of graphs demonstrates the poorer prognosis in terms of disease-specific (DSS), recurrence-free (RFS), and distant recurrence-free survival (DRFS) of patients with progression of disease (PD) after neoadjuvant chemotherapy compared to those with stable disease (SD) or partial response (PR). Middle row shows the protective effect of adjuvant radiotherapy (RT) and chemoradiotherapy (CRT) on local recurrence-free survival (LRFS). Of note, only CRT showed an effect on DSS at univariate analysis. Bottom row shows the absence of a relevant effect of margin status on prognosis in patients receiving neoadjuvant CT. P-value refers to log-rank test (see **Tables S3**–**S6** for multivariable-adjusted significance). CT, adjuvant chemotherapy; R0, clear margins; R1, involved margins.

Adjuvant and neoadjuvant therapies have been confirmed to be of utmost importance in determining and predicting outcomes. Response to neoadjuvant ChT was associated with OS, DSS, and RFS independently of histology, locoregional extension, type of surgery, margin status, and type of adjuvant treatment (**Tables S3–S5**). In particular, patients with PD had a significantly worse prognosis than those with SD or PR following neoadjuvant ChT (**Figure 8**). Several studies from the University of Texas MD Anderson Cancer Center demonstrated the prognostic effect of response to neoadjuvant ChT in single-histology SNC series (20, 27, 28). Of note, in our series, adjuvant ChT-RT showed a remarkable positive effect on RFS and LRFS (**Figure 8**), which was, however, minimized in multivariable analysis, where adjuvant RT and ChT-RT had a similar positive impact on RFS and LRFS irrespective of histology, locoregional extension, type of surgery, margin status, and previous ChT (**Tables S5–S6**). This finding reinforces the belief that treatment of most SNC should be multimodal. Being independently associated with OS, DSS, RFS, and LRFS, margin status was confirmed as a relevant prognostic factor. The finding that margin status lost its prognostic effect on patients treated with neoadjuvant ChT is of particular interest (**Tables S3–S6**). A possible explanation might be related to a non-concentric response of SNC to ChT, as observed in other cancers (29). This would imply that the assessment of margins at definitive pathology is not a reliable estimate of microscopic residual disease. Such a hypothesis, while based on a small number of patients, might suggest that the value of classical prognosticators of SNC is undermined in subjects receiving neoadjuvant therapies and warrants a systematic reappraisal of prognostic factors in these patients. However, before generalizing this finding, one should consider that it was based on patients sent for surgery after neoadjuvant ChT, which includes a majority of cases with poor response to neoadjuvant therapy and might thereby be not representative of all SNC.

### Machine-Learning-Based Classification of Sinonasal Carcinomas

Three machine-learning-generated experimental classifications of SNC based on pathological features and locoregional extension were generated to test the main hypothesis of the study, which was that reorganization of clinicopathological information could improve the prediction of prognosis and chemoradiosensitivity. Each classification was compared to the respective gold standard method to describe sinonasal cancers, namely the WHO (1) and TNM classifications (13) for pathological diagnosis and description of locoregional extension, respectively. A comparison was first performed on a prognostic basis and showed that classification #2 better predicted OS than the WHO classification, whereas classification #1 could not be included in the comparison as there were no prognostic outcomes affected by both the WHO classification and classification #1 at multivariable analyses. Prognostic segregation was also better for classification #2 compared to the WHO classification, with 37.2 and 9.0% of patients being classified in the poor-prognosis category(ies), respectively. As opposed to classification #1, classification #2 was applicable *a priori*, which means that criteria to apply this classification to an external series could be found, as reported in **Figure 6**. However, two main drawbacks of this classification should be highlighted: First, criteria to designate a cancer with one or another differentiation were established arbitrarily, based on the common interpretation of some morphological and (immuno)histochemical findings (**Table 1**); second, immunohistochemistry, which is supposed to substantially aid in unveiling nuances of differentiation in a given cancer, was not systematically applied, as immunostaining was dictated by case-specific needs to achieve a diagnosis. Thus, not all cancers underwent the same set of immunostaining. Based on these findings, reorganization of pathological information, with special reference to those related to tumor differentiation, may help in improving the ability to predict outcomes of SNC-patients. However, classification #2 has been used only as a research means to test a scientific hypothesis and requires optimization and external validation prior to being proposed as an alternative prognostic tool.

Classification #3 assessed the locoregional extension of SNC and performed better than TNM classification in terms of OS, DSS, RFS, and LRFS prediction. However, the absence of *a priori* applicability prevents it from being proposed as an alternative to TNM classification. Moreover, TNM-based clustering of tumors provided better prognostic segregation. The main difference between classification #3 and TNM lies in the fact that in the former, the category is assigned by simultaneously considering the status of involvement of several sinonasal and skull base structures, whereas the latter classifies a tumor based on the infiltrated structure pertaining to the highest T category. The fact that the first method provided better prediction of several survival outcomes should prompt investigation of tumor extension in a more multidimensional fashion when T category assignment criteria are revised for the next TNM Edition. For instance, a score-based assignment based on the evaluation of tumor extension along the 6 vectors of possible growth (i.e., anterior, posterior, inferior, superior, medial, and lateral) could be considered.

Of note, the prognostic value of the experimental classification used herein should be considered in view of the unsupervised methodology of machine learning. Each classifications has been developed based on non-prognostic information, and prognostic outcomes (i.e., DSS and LRFS) were employed only to select the best among alternative clustering strategies developed blindly with respect to prognosis. This method minimizes the risk of overfitting.

### Unpredictability of Response to Chemotherapy and Radiotherapy

Reliable prediction of sensitivity to ChT and/or RT is an unmet need in the field of sinonasal oncology. Amit et al. recently demonstrated that sensitivity to ChT-RT can be based on response to neoadjuvant ChT in SNUC, at the cost of a 60% rate of ChT-related grade 3–4 adverse events (20). The same group also found that a 34-gene signature predicted the response to neoadjuvant ChT in SNUC, thus paving the way towards molecular biology-based selection of locoregional treatment, which would have the potential benefit of avoiding neoadjuvant ChT-related toxicity (30). Different from SNUC, chemoselection is not effective in other SNC such as SCC (28). Since SNUC represents a minority of SNC, there is an evident need for predictive tools to identify responders to non-surgical treatment. This would, in fact, save potential responders the morbidity of invasive surgeries such as open maxillectomy and endoscopic-assisted craniofacial resection, which were performed in more than half of the patients in the present series (83/145, 57.2%). Our analysis showed that PNI and pagetoid growth affected chemoradiosensitivity independently of other factors. PNI was associated with resistance to non-surgical therapies, whereas pagetoid growth was associated with increased chemoradiosensitivity. However, this observation has limited value from a predictive perspective: first, PNI and pagetoid growth were found at definitive histological examination after surgery, and one could argue that they might have been undetectable at pre-treatment biopsy; second, even if pagetoid growth was unprecedently reported in SNC in the present study (**Figure 3D**), this pattern of local extension is rather rare (7/145 cases, 4.8%). Pagetoid growth refers to the tendency of cancer cells to spread through the epithelium, thus representing a distinct escape route along the superficial aspect of the sinonasal tract compared to invasion of subepithelial tissues. This pattern was observed in 3 SNCNOS, 2 SCC, and 2 SNUC. Two cases were recurrent, and 3 had been treated with neoadjuvant ChT (of which 1 was a recurrent SNCNOS), thus excluding that this pattern represents an artifact induced by previous treatments (as 3 patients were treatment-naïve). PNI was observed in 44 (30.3%) patients, consistent with the findings of Gil et al. (22% in SCC, 60% in SNUC, 20% in SNCNOS) (31). The fact that PNI increased chemoradioresistance is consistent with its negative effect on prognosis, as observed in other studies (11, 32). Thus, it is reasonable to surmise that PNI represents a preeminent mechanism of resistance to therapy in SNC. Overall, it can be concluded that pathological features cannot be exploited, not even through machine learning, to infer chemoradiosensitivity of SNC, as also witnessed by the fact that CRF was unable to segregate cancers based on their estimated chemoradiosensitivity. Thus, since genomics- (30) and radiomics-based (33) signatures are efficient in segregating responders from non-responders while avoiding chemoselection, omic analysis of SNC represents the next logical step forward in sinonasal oncology research.

### Limitations of the Study

Besides those already highlighted, the 1) retrospective design of this study, which included only patients treated with surgery as locoregional treatment, is the major limitation. This was imposed by the need for accurate pathological analysis of each case (that is not available in patients receiving a primary RT-based treatment), but, at the same time, it creates a considerable selection bias. In fact, some chemoradiosensitive cancers that initially responded to neoadjuvant ChT were treated with definitive ChT-RT, thus preventing inclusion in this study. 2) Given the rarity of SNC, both primary and recurrent cases were included. Removing recurrent cases would have meant decreasing the size of the series to a point of non-usability for this study. Since presentation did not significantly impact on survival, with prognosis being the first term of comparison between classifications, then the tradeoff between dramatically reducing the series size and accepting the non-/poorly-impacting approximation of including non-primary cases was considered in favor of this scientific policy. Of note, the same strategy has been adopted by several other research groups with a high reputation in the field of sinonasal cancer (34–41). However, despite there exists no sound and univocal evidence on the fact that recurrent SNC bear worse prognosis compared to primary SNC, a sufficiently large series of non-recurrent SNC would imply reducing the risk for confounders and bias. 3) Selection of staining methods to make diagnosis was dictated by case-specific needs and constraints, thus being non-systematic as an unavoidable consequence of the long inclusion period. 4) Lacking a blind re-evaluation by multiple raters, this study does not provide information on inter-rater agreement. 5) The sample size of this single-center series of rare cancers is inherently limited. 6) Even if unsupervised machine learning reduces the risk of overfitting, external validation will be essential to corroborate our findings.

## Conclusions

This study confirmed that SNCs are exceedingly heterogeneous from a histological standpoint. Oncologic outcomes have plateaued since the early 2000s despite the adoption of multi-modal treatment regimens. SNCNOS, namely cancers that cannot be precisely classified as per WHO criteria, represent a non-negligible part of SNC and their prognosis is similar to that of aggressive histologies such as NEC and ID-SNUC. Re-classification of cancers through a machine learning method based on pathological information improved prediction and segregation, thus suggesting that a reappraisal of pathological and biological features of these cancers could be beneficial in terms of prognostic accuracy. However, the response to ChT and/or RT could not be predicted in this series, thus suggesting that other fields of research, such as radiomics and genomics/transcriptomics, should be exploited to identify predictive models. Of note, the classifications presented here were aimed at verifying the hypothesis of the study and are not intended to substitute the standardized method for classifying SNC.

## Data Availability Statement

The raw data supporting the conclusions of this article will be made available by the authors, without undue reservation.

## Ethics Statement

The studies involving human participants were reviewed and approved by the Comitato Etico degli Spedali Civili di Brescia. Written informed consent for participation was not required for this study in accordance with the national legislation and the institutional requirements.

## Author Contributions

Authors contributed to the manuscript as follows: conceptualization: MF, DM, AS, VR, RM, PB, AD, and PN. Methodology: MF. Software: MF. Formal analysis: DM and MR. Data curation: MF, VR, TG, MT, ST, LA, SB, and AB. Writing of original draft: MF. Manuscript review and editing: DM, CP, PB, AD, and PN. Supervision: RM, PB, CP, AD, and PN. All authors listed have made a substantial, direct, and intellectual contribution to the work and approved it for publication.

## Conflict of Interest

The authors declare that the research was conducted in the absence of any commercial or financial relationships that could be construed as a potential conflict of interest.

## Publisher’s Note

All claims expressed in this article are solely those of the authors and do not necessarily represent those of their affiliated organizations, or those of the publisher, the editors and the reviewers. Any product that may be evaluated in this article, or claim that may be made by its manufacturer, is not guaranteed or endorsed by the publisher.
