# Supplementary material for: Does Reorganization of Clinicopathological Information Improve Prognostic Stratification and Prediction of Chemoradiosensitivity in Sinonasal Carcinomas? A Retrospective Study on 145 Patients
Source: Front Oncol. 2022 Jun 3;12:799680. doi: 10.3389/fonc.2022.799680 (PMC9203696; doi:10.3389/fonc.2022.799680)
Supplement: Supplementary file 1 [file DataSheet_1.docx]

| **Information group** | **Information** | **Description (categories)** |
| --- | --- | --- |
| Demographics | Age at surgery | As continuous variable in years |
|  | Gender | - Male - Female |
| Oncologic history | Presentation | - Primary - Recurrent |
|  | Previous treatment | - Surgery - Radiotherapy - Chemotherapy - Combination thereof |
| Treatment | Type of surgery | - Endoscopic resection - Endoscopic resection with transnasal craniectomy - Cranioendoscopic resection - Open maxillectomy - Endoscopic-assisted craniofacial resection |
|  | Neoadjuvant chemotherapy | - No - Yes |
|  | Response to neoadjuvant chemotherapy as per RECIST ver. 1.1 criteria | - Complete response - Partial response - Stable disease - Progression of disease |
|  | Adjuvant treatment | - None - Radiotherapy - Chemoradiotherapy |
| General pathological features | Histology according to WHO 7^th^ Ed. classification | - Squamous cell carcinoma - Sinonasal undifferentiated carcinoma without molecular identifier - SMARCB1/INI1-deficient carcinoma - High-grade non-intestinal-type adenocarcinoma - Neuroendocrine carcinoma - Sinonasal carcinoma not otherwise specified |
|  | Preeminent grade of differentiation | - Well differentiated - Moderately differentiated - Poorly differentiated - Undifferentiated |
|  | Worst grade of differentiation | - Well differentiated - Moderately differentiated - Poorly differentiated - Undifferentiated |
|  | Margin status | - Clear - Involved |
| Cytomorphological information | Squamous-differentiated cell | - No - Yes |
|  | Basaloid-differentiated cells | - No - Yes |
|  | Glandular-differentiated cells | - No - Yes |
|  | Mesenchymal-differentiated cells | - No - Yes |
|  | Type of mesenchymal-differentiated cells | - Spindle - Rhabdoid - Osteoblastoid - Combination thereof |
|  | Keratinization | - No - Yes |
|  | Cellular pleomorphism | - No - Yes |
|  | Nuclear pleomorphism | - No - Yes |
|  | Nucleolar prominence | - No - Yes |
|  | Atypical mitoses | - No - Yes |
|  | Neoplastic necrosis | - No - Yes |
|  | Verrucous hyperplasia | - No - Yes |
|  | Nucleus/cytoplasm ratio | - Low - Intermediate - High |
| Histomorphological and local invasion-related information | Pattern of growth | - Solid - Papillary - Tubular - Lobular - Cribriform - Transitional-like - Pagetoid - Combination thereof |
|  | Perineural invasion | - No - Yes |
|  | Lymphovascular invasion | - No - Yes |
|  | Infiltrative pattern-bone invasion | - No - Yes |
| Differentiation  (see Table 1) | Squamous | - No - Yes |
|  | Glandular | - No - Yes |
|  | Neuroendocrine | - No - Yes |
|  | Mesenchymal | - No - Yes |
|  | Embryonal | - No - Yes |
|  | Neural | - No - Yes |
| Locoregional extension | Pathological T category | - T1 - T2 - T3 - T4a - T4b |
|  | Orbital involvement | - No - Yes |
|  | Bony skull base involvement | - No - Yes |
|  | Dura mater involvement | - No - Yes |
|  | Masticator space involvement | - No - Yes |
|  | Parapharyngeal space involvement | - No - Yes |
|  | Facial soft tissues | - No - Yes |
|  | Sphenoidal sinus (walls) | - No - Yes |
|  | Frontal sinus (walls) | - No - Yes |
|  | Nasopharynx (walls) | - No - Yes |
|  | Nodal metastasis | - No - Yes |
| Immunohistochemical and nucleic acid-based test information | See Table S2 | See Table S2 |

**Table S1.** List of information retrospectively acquired in the present study.

| **Staining type** | **Staining** | |
| --- | --- | --- |
| **Histochemistry** | 1. PAS: 3+, 8- | |
| **Immunohistochemistry** | 1. Low molecular weight cytokeratin (CK5, CK6, CAM 5.2): 20+, 2- 2. High molecular weight cytokeratin (CK8, CK18, CK903): 3+, 1- 3. Pancytokeratin: 43+, 3- 4. CK7: 16+, 18- 5. CK14: 2+, 3- 6. CK19: 3+, 1- 7. CK20: 7+, 20- 8. CK-AE1/AE3: 5+, 4- 9. p63: 22+, 15- 10. p40: 12+, 4- 11. EMA/MUC1: 12+, 8- 12. CD56: 15+, 8- 13. Synaptophysin: 19+, 19- 14. Chromogranin A: 16+, 25- 15. NSE: 3+, 2- 16. GFAP: 1+, 7- 17. Calretinin: 4+, 3- 18. TTF-1: 4+, 6- 19. Vimentin: 3+, 4- 20. Smooth muscle alfa-actin: 6+, 10- 21. Calponin: 3+, 4- 22. Myogenin: 2+, 5- 23. Desmin: 4+, 10- 24. Claudin: 1+ 25. SOX-10: 2+, 4- 26. SOX-2: 1- 27. CD117: 2+, 1- 28. S100: 7+, 37- 29. ERG: 1+, 1- 30. EGFR: 2- 31. HER2: 1- 32. DOG-1: 1+ 33. BCL-2: 1+, 1- | 1. GATA3: 1- 2. PLAP: 1- 3. CD2: 1- 4. CD3: 4- 5. CD5: 1- 6. CD10: 1- 7. CD20: 4- 8. CD21: 1- 9. CD30: 6- 10. CD31: 4- 11. CD34: 5- 12. CD35: 1- 13. CD45: 5- 14. CD68: 1+, 1- 15. CD79: 1- 16. CD99: 1+, 10- 17. CD138: 2- 18. FXIIIa: 1+ 19. LMP-1: 1- 20. Inhibin: 1- 21. MYC: 1+ 22. AR: 2- 23. ER: 1+, 4- 24. PR: 2- 25. HMB-45: 9- 26. MART-1: 1+, 7- 27. p53: 2+ 28. p16: 5+, 4- 29. NUT: 1- 30. INI1: 8+, 3- 31. CEA: 1+, 2- 32. Calcitonin: 2- 33. PTH: 2- 34. Polyomavirus: 3- |
| **Nucleic acid** | 1. EBER: 1+, 15- 2. HPV-DNA: 3- | |

**Table S2.** List of histochemical, immunohistochemical, and nucleic acid-based staining performed over the inclusion period. The absolute frequency of positive (+) and negative (-) results is reported.

| **Overall survival** | **Model #1** | **Model #2** | **Model #3*** |
| --- | --- | --- | --- |
| **Parameters included in the model** | - Type of surgery - Histology - Class of locoregional extension** - Margin status | - Type of surgery - Histology - Class of locoregional extension** - Margin status - Type of adjuvant treatment | - Type of surgery - Histology - Class of locoregional extension** - Margin status - Response to neoadjuvant ChT - Type of adjuvant treatment |
| **Covariates (RR, p-value)** | Type of surgery:   - CER: *REF* - ER: 0.69, p=0.792 - **ERTC: 0.16, p=0.002** - OM: 1.76, p=0.295 - (EA)CFR: 1.57, p=0.358 | Type of surgery:   - CER: *REF* - ER: 0.74, p=0.610 - **ERTC: 0.18, p=0.011** - OM: 2.04, p=0.212 - (EA)CFR: 1.78, p=0.280 | Type of surgery:   - CER: *REF* - ER: N.C., p=0.999 - ERTC: N.C., p=0.999 - OM: N.C., p=0.999 - (EA)CFR: N.C., p=0.999 |
|  | Histology:   - HG-NITAC: *REF* - SCC: 1.07, p=0.920 - ***SNCNOS: 3.17, p=0.092*** - **NEC: 15.09, p=0.002** - SNUC: 1.35, p=0.800 - **ID-SNUC: 8.57, p=0.022** | Histology:   - HG-NITAC: *REF* - SCC: 0.77, p=0.700 - SNCNOS: 2.10, p=0.314 - **NEC: 10.90, p=0.013** - SNUC: 1.08, p=0.956 - **ID-SNUC: 7.10, p=0.047** | Histology:   - HG-NITAC: *REF* - SCC: 1.22, p=0.918 - ***SNCNOS: 48.09, p=0.071*** - **NEC: 166.51, p=0.043** - SNUC: N.C., p=0.998 - **ID-SNUC: 2.42*10^3^, p=0.034** |
|  | Class of locoregional extension:   - Class 1: *REF* - Class 2: 1.12, p=0.801 - **Class 3: 5.39, p=0.001** - **Class 4: 3.28, p=0.004** - **Class 5: 7.07, p<0.0001** | Class of locoregional extension:   - Class 1: *REF* - Class 2: 1.09, p=0.843 - **Class 3: 5.75, p=0.001** - **Class 4: 3.37, p=0.003** - **Class 5: 6.32, p=0.0003** | Class of locoregional extension:   - Class 1: *REF* - Class 2: 11.20, p=0.185 - Class 3: 0.67, p=0.848 - **Class 4: 124.04, p=0.046** - Class 5: 2.09*10^3^, p=0.203 |
|  |  |  | Margin status:   - R0: *REF* - R1: 1.59, p=0.831 |
|  |  | Margin status:   - R0: *REF* - ***R1: 1.81, p=0.053*** | Response to neoadjuvant ChT:   - PD: *REF* - **SD: 0.01, p=0.009** - **PR: 0.01, p=0.026** |
|  | Margin status:   - R0: *REF* - **R1: 1.83, p=0.047** | Type of adjuvant treatment:   - None: *REF* - RT: 0.61, p=0.115 - RT-ChT: 0.38, p=0.102 - ChT: 0.69, p=0.598 | Type of adjuvant treatment:   - None: *REF* - RT: 0.39, p=0.593 - RT-ChT: 0.43, p=0.552 - ChT: N.C., p=0.998 |
| **Performance of the model** | - C-index: 0.589 - AIC: 527 - BIC: 557 - NPR: 0.720 | - C-index: 0.598 - AIC: 529 - BIC: 566 - NPR: 0.773 | - C-index: 0.558 - AIC: 105 - BIC: 120 - NPR: 0.978 |

**Table S3.** Multivariable models created to analyze prognostic factors in terms of overall survival. *Model #3 refers only to the subgroup of patients treated with neoadjuvant chemotherapy (ChT). **Class of locoregional extension is attributed according to classification #3. AIC, Akaike information criterion; BIC, Bayesian information criterion; C-index, concordance index; CER, cranioendoscopic resection; (EA)CFR, craniofacial resection with possible endoscopic assistance; ER, endoscopic resection; ERTC, endoscopic resection with transnasal craniectomy; HG-NITAC, high-grade non-intestinal-type adenocarcinoma; ID-SNUC, INI1/SMARCB1-deficient sinonasal undifferentiated carcinoma; N.C., not calculable; NEC, neuroendocrine carcinoma; NPR, Nagelkerke pseudo-R^2^; OM, open maxillectomy; PD, progression of disease; PR, partial response; R0, clear margins; R1, microscopically involved margins; REF, reference category; RR, relative risk; RT, radiotherapy; SCC, squamous cell carcinoma; SD, stable disease; SNCNOS, sinonasal carcinoma not otherwise specified; SNUC, sinonasal undifferentiated carcinoma. Significance (p<0.05) is highlighted in **bold**. Categories approaching significance (0.05≤p<0.10) are highlighted in ***bold italics***.

| **Disease-specific survival** | **Model #1** | **Model #2** | **Model #3*** |
| --- | --- | --- | --- |
| **Parameters included in the model** | - Type of surgery - Histology - Class of locoregional extension** - Margin status | - Type of surgery - Histology - Class of locoregional extension** - Margin status - Type of adjuvant treatment | - Type of surgery - Histology - Class of locoregional extension** - Margin status - Response to neoadjuvant ChT - Type of adjuvant treatment |
| **Covariates (RR, p-value)** | Type of surgery:***   - CER: *REF* - ER: 0.65, p=0.501 - **ERTC: 0.14, p=0.002** - OM: 2.29, p=0.146 - (EA)CFR: 2.24, p=0.121 | Type of surgery:***   - CER: *REF* - ER: 0.64, p=0.490 - **ERTC: 0.19, p=0.023** - ***OM: 2.94, p=0.080*** - ***(EA)CFR: 2.86, p=0.071*** | Type of surgery:   - CER: *REF* - ER: N.C., p=0.999 - ERTC: N.C., p=0.999 - OM: N.C., p=0.999 - (EA)CFR: N.C., p=0.999 |
|  | Histology:***   - HG-NITAC: *REF* - SCC: 1.80, p=0.449 - **SNCNOS: 5.34, p=0.039** - **NEC: 30.40, p=0.001** - SNUC: 2.77, p=0.423 - **ID-SNUC: 10.83, p=0.022** | Histology:***   - HG-NITAC: *REF* - SCC: 1.13, p=0.878 - SNCNOS: 3.00, p=0.208 - **NEC: 16.43, p=0.011** - SNUC: 1.43, p=0.805 - **ID-SNUC: 8.98, p=0.046** | Histology:   - HG-NITAC: *REF* - SCC: 1.32, p=0.896 - SNCNOS: 38.86, p=0.124 - **NEC: 913.86, p=0.021** - SNUC: N.C., p=0.999 - **ID-SNUC: 3.67*10^3^, p=0.036** |
|  | Class of locoregional extension:***   - Class 1: *REF* - Class 2: 0.73, p=0.504 - **Class 3: 6.33, p=0.001** - **Class 4: 2.44, p=0.042** - **Class 5: 7.11, p=0.0001** | Class of locoregional extension:***   - Class 1: *REF* - Class 2: 0.73, p=0.491 - **Class 3: 6.73, p=0.001** - **Class 4: 2.50, p=0.035** - **Class 5: 5.91, p=0.001** | Class of locoregional extension:   - Class 1: *REF* - Class 2: 12.19, p=0.217 - Class 3: 36.44, p=0.208 - ***Class 4: 87.09, p=0.072*** - Class 5: 471.77, p=0.215 |
|  |  |  | Margin status:   - R0: *REF* - R1: 1.09, p=0.969 |
|  |  | Margin status:***   - R0: *REF* - **R1: 2.29, p=0.014** | Response to neoadjuvant ChT:   - PD: *REF* - **SD: 0.01, p=0.014** - **PR: 0.01, p=0.049** |
|  | Margin status:***   - R0: *REF* - **R1: 2.44, p=0.008** | Type of adjuvant treatment:****   - None: *REF* - ***RT: 0.58, p=0.093*** - ***RT-ChT: 0.28, p=0.063*** - ChT: 0.97, p=0.962 | Type of adjuvant treatment:   - None: *REF* - RT: 0.04, p=0.105 - ***RT-ChT: 0.05, p=0.060*** - ChT: N.C., p=0.999 |
| **Performance of the model** | - C-index: 0.591 - AIC: 467 - BIC: 495 - NPR: 0.764 | - C-index: 0.608 - AIC: 467 - BIC: 502 - NPR: 0.794 | - C-index: 0.541 - AIC: 94 - BIC: 108 - NPR: 0.979 |

**Table S4.** Multivariable models created to analyze prognostic factors in terms of disease-specific survival. *Model #3 refers only to the subgroup of patients treated with neoadjuvant chemotherapy (ChT). **Class of locoregional extension is attributed according to classification #3. ***Significance confirmed at competing risk analysis. AIC, Akaike information criterion; BIC, Bayesian information criterion; C-index, concordance index; CER, cranioendoscopic resection; (EA)CFR, craniofacial resection with possible endoscopic assistance; ER, endoscopic resection; ERTC, endoscopic resection with transnasal craniectomy; HG-NITAC, high-grade non-intestinal-type adenocarcinoma; ID-SNUC, INI1/SMARCB1-deficient sinonasal undifferentiated carcinoma; N.C., not calculable; NEC, neuroendocrine carcinoma; NPR, Nagelkerke pseudo-R^2^; OM, open maxillectomy; PD, progression of disease; PR, partial response; R0, clear margins; R1, microscopically involved margins; REF, reference category; RR, relative risk; RT, radiotherapy; SCC, squamous cell carcinoma; SD, stable disease; SNCNOS, sinonasal carcinoma not otherwise specified; SNUC, sinonasal undifferentiated carcinoma. Significance (p<0.05) is highlighted in **bold**. Categories approaching significance (0.05≤p<0.10) are highlighted in ***bold italics***.

| **Recurrence-free survival** | **Model #1** | **Model #2*** |
| --- | --- | --- |
| **Parameters included in the model** | - Type of surgery - Histology - Class of locoregional extension** - Margin status - Type of adjuvant treatment | - Type of surgery - Histology - Class of locoregional extension** - Margin status - Response to neoadjuvant ChT - Type of adjuvant treatment |
| **Covariates (RR, p-value)** | Type of surgery:   - CER: *REF* - ER: 1.25, p=0.719 - **ERTC: 0.25, p=0.030** - ***OM: 2.81, p=0.086*** - (EA)CFR: 2.12, p=0.183 | Type of surgery:   - CER: *REF* - ER: N.C., p=N.C. - ERTC: 0.21, p=0.535 - **OM: 58.27, p=0.037** - (EA)CFR: 2.59, p=0.476 |
|  | Histology:   - HG-NITAC: *REF* - SCC: 1.10, p=0.892 - SNCNOS: 2.48, p=0.208 - **NEC: 11.12, p=0.012** - SNUC: 3.41, p=0.326 - ID-SNUC: 3.72, p=0.180 | Histology:   - HG-NITAC: *REF* - SCC: 1.64, p=0.753 - SNCNOS: 9.09, p=0.192 - **NEC: 346.41 p=0.010** - SNUC: N.C., p=0.999 - **ID-SNUC: 884.31, p=0.008** |
|  | Class of locoregional extension:   - Class 1: *REF* - Class 2: 1.08, p=0.855 - **Class 3: 9.16, p<0.0001** - **Class 4: 2.86, p=0.012** - **Class 5: 6.25, p=0.0003** | Class of locoregional extension:   - Class 1: *REF* - ***Class 2: 7.17, p=0.081*** - ***Class 3: 72.73, p=0.093*** - ***Class 4: 19.02, p=0.069*** - Class 5: 5.32, p=0.391 |
|  |  | Margin status:   - R0: *REF* - R1: 0.25, p=0.206 |
|  | Margin status:   - R0: *REF* - **R1: 2.19, p=0.009** | Response to neoadjuvant ChT:   - PD: *REF* - **SD: 0.06, p=0.018** - PR: 0.23, p=0.202 |
|  | Type of adjuvant treatment:   - None: *REF* - **RT: 0.47, p=0.015** - ***RT-ChT: 0.38, p=0.066*** - ChT: 1.81, p=0.395 | Type of adjuvant treatment:   - None: *REF* - **RT: 0.04, p=0.039** - RT-ChT: 0.12, p=0.138 - ChT: 1.59, p=0.849 |
| **Performance of the model** | - C-index: 0.431 - AIC: 579 - BIC: 617 - NPR: 0.781 | - C-index: 0.371 - AIC: 127 - BIC: 146 - NPR: 0.978 |

**Table S5.** Multivariable models created to analyze prognostic factors in terms of recurrence-free survival. *Model #2 refers only to the subgroup of patients treated with neoadjuvant chemotherapy (ChT). **Class of locoregional extension is attributed according to classification #3. AIC, Akaike information criterion; BIC, Bayesian information criterion; C-index, concordance index; CER, cranioendoscopic resection; (EA)CFR, craniofacial resection with possible endoscopic assistance; ER, endoscopic resection; ERTC, endoscopic resection with transnasal craniectomy; HG-NITAC, high-grade non-intestinal-type adenocarcinoma; ID-SNUC, INI1/SMARCB1-deficient sinonasal undifferentiated carcinoma; N.C., not calculable; NEC, neuroendocrine carcinoma; NPR, Nagelkerke pseudo-R^2^; OM, open maxillectomy; PD, progression of disease; PR, partial response; R0, clear margins; R1, microscopically involved margins; REF, reference category; RR, relative risk; RT, radiotherapy; SCC, squamous cell carcinoma; SD, stable disease; SNCNOS, sinonasal carcinoma not otherwise specified; SNUC, sinonasal undifferentiated carcinoma. Significance (p<0.05) is highlighted in **bold**. Categories approaching significance (0.05≤p<0.10) are highlighted in ***bold italics***.

| **Local recurrence-free survival** | **Model #1** | **Model #2** | **Model #3*** |
| --- | --- | --- | --- |
| **Parameters included in the model** | - Type of surgery - Class of locoregional extension** - Margin status - Type of adjuvant treatment - Previous ChT (neoadjuvant ChT excluded) | - Type of surgery - Histology - Class of locoregional extension** - Margin status - Type of adjuvant treatment - Previous ChT (neoadjuvant ChT excluded) | - Type of surgery - Class of locoregional extension** - Margin status - Type of adjuvant treatment - Response to neoadjuvant ChT - Previous ChT (neoadjuvant ChT excluded) |
| **Covariates (RR, p-value)** | Type of surgery:***   - CER: *REF* - ER: 2.28, p=0.313 - ERTC: 0.58, p=0.450 - ***OM: 4.05, p=0.056*** - ***(EA)CFR: 3.35, p=0.074*** | Type of surgery:***   - CER: *REF* - ER: 1.86, p=0.421 - ERTC: 0.29, p=0.116 - **OM: 4.53, p=0.037** - (EA)CFR: 2.83, p=0.123 | Type of surgery:   - CER: *REF* - ER: - - ERTC: 0.49, p=0.770 - OM: 1.50, p=0.812 - (EA)CFR: 1.94, p=0.603 |
|  | Class of locoregional extension:***   - Class 1: *REF* - Class 2: 1.36, p=0.551 - **Class 3: 11.45, p=0.0002** - **Class 4: 5.33, p=0.0006** - **Class 5: 11.44, p<0.0001** | Histology:   - HG-NITAC: *REF* - SCC: 0.91, p=0.912 - SNCNOS: 1.41, p=0.715 - ***NEC: 8.91 p=0.078*** - SNUC: 4.91, p=0.250 - ID-SNUC: 1.60, p=0.694 | Class of locoregional extension:   - Class 1: *REF* - Class 2: 3.01, p=0.397 - Class 3: 3.87, p=0.449 - Class 4: 3.07, p=0.431 - Class 5: 1.03, p=0.989 |
|  | Margin status:***   - R0: *REF* - **R1: 2.16, p=0.017** | Class of locoregional extension:***   - Class 1: *REF* - Class 2: 1.22, p=0.706 - **Class 3: 14.03, p<0.0001** - **Class 4: 4.52, p=0.002** - **Class 5: 13.67, p<0.0001** | Margin status:   - R0: *REF* - R1: 1.02, p=0.978 |
|  |  |  |  |
|  | Type of adjuvant treatment:***   - None: *REF* - **RT: 0.28, p=0.0003** - **RT-ChT: 0.15, p=0.015** - ChT: 3.19, p=0.142 | Margin status:***   - R0: *REF* - **R1: 2.32, p=0.017** | Type of adjuvant treatment:   - None: *REF* - **RT: 0.04, p=0.039** - ***RT-ChT: 0.07, p=0.052*** - ChT: 0.18, p=0.524 |
|  |  | Type of adjuvant treatment:***   - None: *REF* - **RT: 0.28, p=0.0004** - **RT-ChT: 0.16, p=0.020** - ChT: 1.72, p=0.497 | Response to neoadjuvant ChT:   - PD: *REF* - SD: 0.43, p=0.443 - PR: 0.80, p=0.830 |
|  | Previous ChT:   - No: *REF* - Yes: 1.39, p=0.314 | Previous ChT:   - No: *REF* - Yes: 1.15, p=0.708 | Previous ChT:   - No: *REF* - Yes: 0.55, p=0.507 |
| **Performance of the model** | - C-index: 0.570 - AIC: 437 - BIC: 463 - NPR: 0.720 | - C-index: 0.576 - AIC: 438 - BIC: 473 - NPR: 0.791 | - C-index: 0.475 - AIC: 96 - BIC: 105 - NPR: 0.962 |

**Table S6.** Multivariable models created to analyze prognostic factors in terms of local recurrence-free survival. *Model #3 refers only to the subgroup of patients treated with neoadjuvant chemotherapy (ChT). **Class of locoregional extension is attributed according to classification #3. ***Significance confirmed at competing risk analysis (*i.e.*, significant difference of local recurrence-specific events with non-significant difference of informative censorships). AIC, Akaike information criterion; BIC, Bayesian information criterion; C-index, concordance index; CER, cranioendoscopic resection; (EA)CFR, craniofacial resection with possible endoscopic assistance; ER, endoscopic resection; ERTC, endoscopic resection with transnasal craniectomy; HG-NITAC, high-grade non-intestinal-type adenocarcinoma; ID-SNUC, INI1/SMARCB1-deficient sinonasal undifferentiated carcinoma; N.C., not calculable; NEC, neuroendocrine carcinoma; NPR, Nagelkerke pseudo-R^2^; OM, open maxillectomy; PD, progression of disease; PR, partial response; R0, clear margins; R1, microscopically involved margins; REF, reference category; RR, relative risk; RT, radiotherapy; SCC, squamous cell carcinoma; SD, stable disease; SNCNOS, sinonasal carcinoma not otherwise specified; SNUC, sinonasal undifferentiated carcinoma. Significance (p<0.05) is highlighted in **bold**. Categories approaching significance (0.05≤p<0.10) are highlighted in ***bold italics***.

| **Subdistribution hazard model** | **Disease-specific survival*** | **Local recurrence-free survival**** | **Distant recurrence-free survival***** |
| --- | --- | --- | --- |
| **Parameters included in the model** | - Type of surgery - Histology - Class of locoregional extension**** - Margin status - Type of adjuvant treatment | - Type of surgery - Histology - Class of locoregional extension**** - Margin status - Type of adjuvant treatment - Previous ChT (neoadjuvant ChT excluded) | - Histology - Class of locoregional extension**** - Margin status - Type of adjuvant treatment |
| **Covariates (RR, p-value)** | Type of surgery:   - CER: *REF* - ER: 0.56, p=0.390 - **ERTC: 0.27, p=0.042** - ***OM: 3.42, p=0.051*** - **(EA)CFR: 3.63, p=0.041** | Type of surgery:   - CER: *REF* - ER: 2.43, p=0.270 - ERTC: 0.58, p=0.460 - **OM: 7.21, p=0.001** - **(EA)CFR: 5.25, p=0.011** | Histology:   - HG-NITAC: *REF* - SCC: 1.84 (vs 1.63), p=0.630 (vs p=0.654) - SNCNOS: 2.49 (vs 2.33), p=0.490 (vs p=0.455) - NEC: 5.20 (vs 7.89) p=0.560 (vs p=0.095) - SNUC: N.C. (vs N.C.) - ID-SNUC: 17.57 (vs 40.90), p=0.150 (vs p=0.025) |
|  | Histology:   - HG-NITAC: *REF* - SCC: 1.10, p=0.890 - SNCNOS: 2.68, p=0.200 - **NEC: 9.38 p=0.022** - SNUC: 1.21, p=0.840 - **ID-SNUC: 6.79, p=0.028** | Histology:   - HG-NITAC: *REF* - SCC: 0.63, p=0.570 - SNCNOS: 0.86, p=0.870 - NEC: 2.08 p=0.560 - SNUC: 4.43, p=0.210 - ID-SNUC: 0.76, p=0.780 | Class of locoregional extension:   - Class 1: *REF* - Class 2: 0.93 (vs 1.26), p=0.910 (vs p=0.729) - **Class 3: 4.60 (vs 5.77), p=0.001 (vs p=0.001)** - Class 4: 2.04 (vs 4.04), p=0.431 (vs p=0.024) - Class 5: 0.27 (vs 1.07), p=0.500 (vs p=0.955) |
|  | Class of locoregional extension:   - Class 1: *REF* - Class 2: 0.61, p=0.330 - **Class 3: 5.95, p=0.002** - Class 4: 1.91, p=0.140 - **Class 5: 5.24, p=0.002** | Class of locoregional extension:   - Class 1: *REF* - Class 2: 0.87, p=0.800 - **Class 3: 9.25, p<0.001** - **Class 4: 2.85, p=0.036** - **Class 5: 9.47, p<0.001** | Margin status:   - R0: *REF* - R1: 1.57 (vs 1.68), p=0.300 (vs p=0.234) |
|  |  |  |  |
|  | Margin status:   - R0: *REF* - **R1: 2.66, p=0.010** | Margin status:   - R0: *REF* - **R1: 2.80, p=0.012** | Type of adjuvant treatment:   - None: *REF* - RT: 2.03 (vs 1.30), p=0.200 (vs p=0.603) - RT-ChT: 0.74 (vs 0.44), p=0.710 (p=0.381) - **ChT: 11.88 (vs 8.24), p=0.003 (vs p=0.008)** |
|  | Type of adjuvant treatment:   - None: *REF* - RT: 0.63, p=0.140 - **RT-ChT: 0.28, p=0.041** - ChT: 1.49, p=0.530 | Type of adjuvant treatment:   - None: *REF* - **RT: 0.32, p=0.001** - **RT-ChT: 0.14, p=0.026** - ChT: 1.91, p=0.390 |  |
|  |  | Previous ChT:   - No: *REF* - Yes: 1.42, p=0.280 |  |

**Table S7.** Competing risk multivariable models of disease-specific, local recurrence-free, and distant recurrence-free survival. *To be compared with Model #2 of Table S3. **To be compared with Model #2 of Table S5. ***Relative risk values and p-values are compared with respective values as per Cox proportional hazards model. ****Class of locoregional extension is attributed according to classification #3. CER, cranioendoscopic resection; (EA)CFR, craniofacial resection with possible endoscopic assistance; ER, endoscopic resection; ERTC, endoscopic resection with transnasal craniectomy; HG-NITAC, high-grade non-intestinal-type adenocarcinoma; ID-SNUC, INI1/SMARCB1-deficient sinonasal undifferentiated carcinoma; N.C., not calculable; NEC, neuroendocrine carcinoma; OM, open maxillectomy; R0, clear margins; R1, microscopically involved margins; REF, reference category; RR, relative risk; RT, radiotherapy; SCC, squamous cell carcinoma; SNCNOS, sinonasal carcinoma not otherwise specified; SNUC, sinonasal undifferentiated carcinoma. Significance (p<0.05) is highlighted in **bold**. Categories approaching significance (0.05≤p<0.10) are highlighted in ***bold italics***.

| **Number of AHC classes** | **2** | **3** | **4** | **5** | **6** |
| --- | --- | --- | --- | --- | --- |
| **Inter-class variance** | 0.47 | 0.66 | 0.77 | 0.88 | 0.94 |
| **Intra-class variance** | 1.02 | 0.83 | 0.72 | 0.61 | 0.54 |
| **Class breakdown** | Class 1: 83  Class 2: 62 | Class 1: 83  Class 2: 23  Class 3: 39 | Class 1: 83  Class 2: 23  Class 3: 16  Class 4: 23 | Class 1: 65  Class 2: 23  Class 3: 18  Class 4: 16  Class 5: 23 | Class 1: 65  Class 2: 23  Class 3: 18  Class 4: 16  Class 5: 22  Class 6: 1 |
| **Univariate Cox model performance parameters for DSS** | C-index: 0.225  AIC: 514  BIC: 516  NPR: 0.015 | C-index: 0.258  AIC: 513  BIC: 517  NPR: 0.085 | C-index: 0.274  AIC: 515  BIC: 521  NPR: 0.076 | C-index: 0.313  AIC: 506  BIC: 514  NPR: 0.207 | C-index: 0.300  AIC: 507  BIC: 517  NPR: 0.268 |

**Table S8**. Agglomerative Hierarchical Clustering (AHC) to generate classification #1. AIC, Akaike information criterion; BIC, Bayesian information criterion; C-index, concordance index; DSS, disease-specific survival; NPR, Nagelkerke pseudo-R^2^. The column highlighted in grey was selected to create classification #1.

| **Number of AHC classes** | **2** | **3** | **4** | **5** | **6** |
| --- | --- | --- | --- | --- | --- |
| **Inter-class variance** | 0.24 | 0.37 | 0.44 | 0.48 | 0.52 |
| **Intra-class variance** | 0.43 | 0.30 | 0.23 | 0.19 | 0.15 |
| **Class breakdown** | Class 1: 83  Class 2: 62 | Class 1: 83  Class 2: 24  Class 3: 38 | Class 1: 83  Class 2: 24  Class 3: 16  Class 4: 22 | Class 1: 83  Class 2: 8  Class 3: 16  Class 4: 16  Class 5: 22 | Class 1: 83  Class 2: 8  Class 3: 16  Class 4: 16  Class 5: 15  Class 6: 7 |
| **Frequency of differentiation** | - Class 1:   - S (100.0%)* - Class 2:   - S (50.0%)   - G (56.5%)   - NE (40.3%)   - M (51.6%) | - Class 1:   - S (100.0%)* - Class 2:   - S (100.0%)   - G (75.0%)   - NE (0.0%)   - M (66.7%) - Class 3:   - S (18.0%)   - G (44.7%)   - NE (65.8%)   - M (42.1%) | - Class 1:   - S (100.0%)* - Class 2:   - S (100.0%)   - G (75.0%)   - NE (0.0%)   - M (66.7%) - Class 3:   - S (25.0%)   - G (0.0%)   - NE (100.0%)   - M (25.0%) - Class 4:   - S (13.6%)   - G (77.3%)   - NE (40.9%)   - M (54.5%) | - Class 1:   - S (100.0%)* - Class 2:   - S (100.0%)   - G (100.0%)   - NE (0.0%)   - M (0.0%) - Class 3:   - S (100.0%)   - G (62.5.0%)   - NE (0.0%)   - M (100.0%) - Class 4:   - S (25.0%)   - G (0.0%)   - NE (100.0%)   - M (25.0%) - Class 5:   - S (13.6%)   - G (77.3%)   - NE (40.9%)   - M (54.5%) | - Class 1:   - S (100.0%)* - Class 2:   - S (100.0%)   - G (100.0%)   - NE (0.0%)   - M (0.0%) - Class 3:   - S (100.0%)   - G (62.5.0%)   - NE (0.0%)   - M (100.0%) - Class 4:   - S (25.0%)   - G (0.0%)   - NE (100.0%)   - M (25.0%) - Class 5:   - S (0.0%)   - G (66.7%)   - NE (13.4%)   - M (40.0%) - Class 6:   - S (42.9%)   - G (100.0%)   - NE (100.0%)   - M (85.7%) |
| **Univariate Cox model performance parameters for DSS** | C-index: 0.245  AIC: 513  BIC: 515  NPR: 0.030 | C-index: 0.260  AIC: 514  BIC: 518  NPR: 0.058 | C-index: 0.293  AIC: 515  BIC: 521  NPR: 0.086 | C-index: 0.289  AIC: 517  BIC: 525  NPR: 0.129 | C-index: 0.297  AIC: 519  BIC: 529  NPR: 0.121 |

**Table S9**. Agglomerative Hierarchical Clustering (AHC) to generate classification #2. AIC, Akaike information criterion; BIC, Bayesian information criterion; C-index, concordance index; DSS, disease-specific survival; G, glandular; M, mesenchymal; NE, neuroendocrine; NPR, Nagelkerke pseudo-R^2^; S, squamous. The column highlighted in grey was selected to create classification #2.

| **Number of AHC classes** | **2** | **3** | **4** | **5** | **pT**  **(TNM VIII ed.)** | **Stage**  **(TNM VIII ed.)** |
| --- | --- | --- | --- | --- | --- | --- |
| **Inter-class variance** | 0.19 | 0.30 | 0.37 | 0.43 | - | - |
| **Intra-class variance** | 0.42 | 0.31 | 0.24 | 0.19 | - | - |
| **Class numerosity** | Class 1: 114  Class 2: 31 | Class 1: 64  Class 2: 50  Class 3: 31 | Class 1: 64  Class 2: 32  Class 3: 18  Class 4: 31 | Class 1: 64  Class 2: 32  Class 3: 18  Class 4: 14  Class 5: 17 | pT1: 12  pT2: 16  pT3: 22  pT4a: 43  pT4b: 52 | I: 12  II: 15  III: 21  IVA: 42  IVB: 55 |
| **Univariate Cox model performance parameters for LRFS** | C-index: 0.176  AIC: 513  BIC: 515  NPR: 0.030 | C-index: 0.269  AIC: 514  BIC: 518  NPR: 0.058 | C-index: 0.394  AIC: 455  BIC: 460  NPR: 0.379 | C-index: 0.395  AIC: 452  BIC: 460  NPR: 0.422 | C-index: 0.353  AIC: 460  BIC: 470  NPR: 0.387 | C-index: 0.348  AIC: 460  BIC: 468  NPR: 0.362 |

**Table S10**. Agglomerative Hierarchical Clustering (AHC) to generate classification #3. AIC, Akaike information criterion; BIC, Bayesian information criterion; LRFS, local recurrence-free survival; NPR, Nagelkerke pseudo-R^2^, pT, pathological T category The column highlighted in grey was selected to create classification #3.


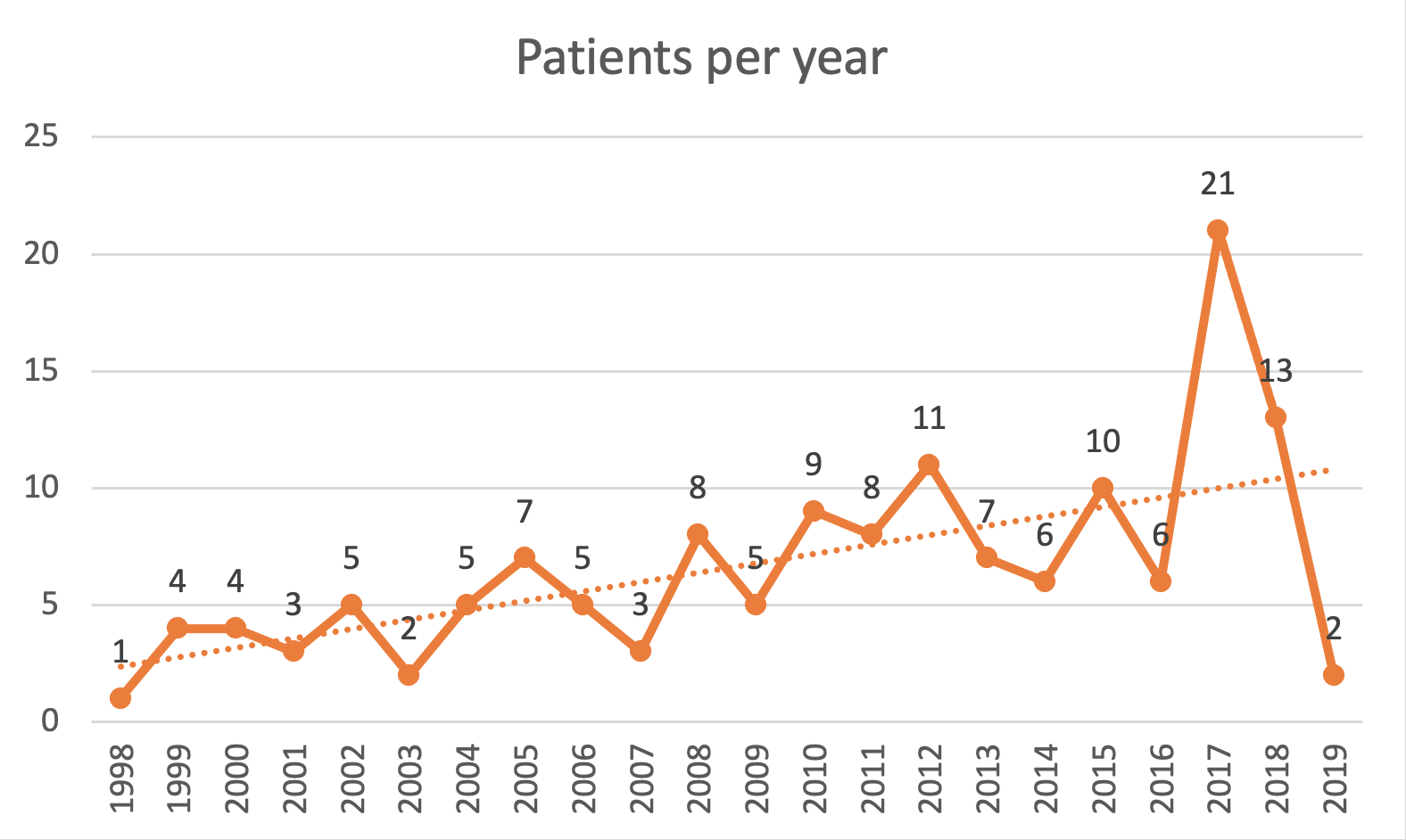


**Figure S1**. The graph shows the count of patients treated per year over the inclusion period.
